# The Multistage Antimalarial Compound Calxinin Perturbates *P. falciparum* Ca^2+^ Homeostasis by Targeting a Unique Ion Channel

**DOI:** 10.3390/pharmaceutics14071371

**Published:** 2022-06-28

**Authors:** Yash Gupta, Neha Sharma, Snigdha Singh, Jesus G. Romero, Vinoth Rajendran, Reagan M. Mogire, Mohammad Kashif, Jordan Beach, Walter Jeske, Poonam, Bernhards R. Ogutu, Stefan M. Kanzok, Hoseah M. Akala, Jennifer Legac, Philip J. Rosenthal, David J. Rademacher, Ravi Durvasula, Agam P. Singh, Brijesh Rathi, Prakasha Kempaiah

**Affiliations:** 1 Infectious Diseases, Mayo Clinic, Jacksonville, FL 32224, USA; gupta.yash1@mayo.edu (Y.G.); durvasula.ravi@mayo.edu (R.D.); 2 Laboratory for Translational Chemistry and Drug Discovery, Department of Chemistry, Hansraj College, University of Delhi, New Delhi 110021, India; nehasharma0024@gmail.com (N.S.); snigdhaantil@gmail.com (S.S.); 3 Stritch School of Medicine, Loyola University Chicago, Chicago, IL 60660, USA; jesus.g.romero.m@gmail.com (J.G.R.); jbeach1@luc.edu (J.B.); wjeske@luc.edu (W.J.); drademacher@luc.edu (D.J.R.); 4 School of Biology, Institute of Experimental Biology, Central University of Venezuela, Caracas 1040, Venezuela; 5 Department of Microbiology, School of Life Sciences, Pondicherry University, Puducherry 605014, India; vinoth.avj@gmail.com; 6 Centre Clinical Research, Kenya Medical Research Institute, Nairobi P.O. Box 54840-00200, Kenya; reaganmoseti@gmail.com (R.M.M.); ogutu6@gmail.com (B.R.O.); akalahoseah@gmail.com (H.M.A.); 7 Infectious Diseases Laboratory, National Institute of Immunology, New Delhi 110067, India; kashif@nii.ac.in (M.K.); singhap@nii.res.in (A.P.S.); 8 Department of Chemistry, Miranda House, University of Delhi, New Delhi 110021, India; poonam.chemistry@mirandahouse.ac.in; 9 Delhi School of Public Health, Institute of Eminence, University of Delhi, New Delhi 110007, India; 10 Department of Biology, Loyola University Chicago, Chicago, IL 60660, USA; skanzok@luc.edu; 11 Department of Medicine, University of California San Francisco, San Francisco, CA 94158, USA; jenny.legac@ucsf.edu (J.L.); philip.rosenthal@ucsf.edu (P.J.R.); 12 Core Imaging Facility and Department of Microbiology and Immunology, Loyola University Chicago, Maywood, IL 60153, USA

**Keywords:** antimalarial, multistage activity, Ca^2+^ homeostasis, transient receptor potential mucolipin like channel, electron microscopy, field isolates

## Abstract

Malaria elimination urgently needs novel antimalarial therapies that transcend resistance, toxicity, and high costs. Our multicentric international collaborative team focuses on developing multistage antimalarials that exhibit novel mechanisms of action. Here, we describe the design, synthesis, and evaluation of a novel multistage antimalarial compound, ‘Calxinin’. A compound that consists of hydroxyethylamine (HEA) and trifluoromethyl-benzyl-piperazine. Calxinin exhibits potent inhibitory activity in the nanomolar range against the asexual blood stages of drug-sensitive (3D7), multidrug-resistant (Dd2), artemisinin-resistant (IPC4912), and fresh Kenyan field isolated *Plasmodium falciparum* strains. Calxinin treatment resulted in diminished maturation of parasite sexual precursor cells (gametocytes) accompanied by distorted parasite morphology. Further, in vitro liver-stage testing with a mouse model showed reduced parasite load at an IC_50_ of 79 nM. A single dose (10 mg/kg) of Calxinin resulted in a 30% reduction in parasitemia in mice infected with a chloroquine-resistant strain of the rodent parasite *P. berghei*. The ex vivo ookinete inhibitory concentration within mosquito gut IC_50_ was 150 nM. Cellular in vitro toxicity assays in the primary and immortalized human cell lines did not show cytotoxicity. A computational protein target identification pipeline identified a putative *P*. *falciparum* membrane protein (*Pf*3D7_1313500) involved in parasite calcium (Ca^2+^) homeostasis as a potential Calxinin target. This highly conserved protein is related to the family of transient receptor potential cation channels (TRP-ML). Target validation experiments showed that exposure of parasitized RBCs (pRBCs) to Calxinin induces a rapid release of intracellular Ca^2+^ from pRBCs; leaving de-calcinated parasites trapped in RBCs. Overall, we demonstrated that Calxinin is a promising antimalarial lead compound with a novel mechanism of action and with potential therapeutic, prophylactic, and transmission-blocking properties against parasites resistant to current antimalarials.

## 1. Introduction

Malaria continues to be a major global health threat and is endemic in over 100 countries, putting more than 3.4 billion people at risk [1]. Despite considerable progress in malaria control and reduction efforts, in 2019, there were an estimated 241 million cases and 627,000 attributable deaths. An increase of 69,000 deaths over the previous year was attributed, in part, to disruptions due to COVID-19 [1,2]. The majority of available antimalarial drugs act against the symptom-causing blood stages of the *Plasmodium* parasites [3], with a high probability of selection for drug-resistant mutants. Artemisinin-based combination therapies (ACTs) are currently the first-line treatments for the deadly *Plasmodium falciparum* (*Pf)* infections. However, the emergence of artemisinin-resistant strains globally in Eastern India, South America (Fr Guiana), and East Africa [4,5,6,7,8,9] challenges this treatment. There is an urgent need for the discovery of new antimalarials, ideally with novel mechanisms of action (MOA) [10,11]. The current interest focuses on the development of antimalarials that can act against multiple parasite life cycle stages. This will enable chemopreventive (against liver stages), therapeutic (against asexual erythrocytic stages), and transmission-blocking (against gametocytes) treatments that will facilitate progress towards the control and eventual elimination of malaria [12].

The selection and design of novel chemical scaffolds for antimalarial drug development remains challenging. The literature and our ongoing research suggest hydroxyethylamine (HEA) and fluorinated piperazine scaffolds as potential starting points for the development of new compounds with potential multistage antimalarial activity. Studies have shown that oral administration of HEA in mouse models results in the clearance of all stages of the *Plasmodium* parasite [13]. These characteristics highlight the HEA scaffold as a promising component for new antimalarial drugs [14]. A newly designed compound from the HEA series, named Calxinin also contains a trifluoromethyl-benzyl-piperazine group The advantage of having a trifluoromethyl group is to stabilize the binding of the main pharmacophore and protect the molecule from degradation [15,16,17].

The mode of synthesis of Calxinin is being published separately (US application#: 17/347720; Indian patent application #: 202011056923/Dated Dec 292020). Here, we report on Calxinin’s antiplasmodial activity and propose an MOA. Calxinin exhibits potent activity against drug-resistant reference strains, as well as clinical *Pf* strains from malaria endemic regions. It is also active against *P. berghei* (*Pb*) in a mouse model, and liver, erythrocyte, and mosquito (ookinete) stages. There is also a significant *Pf* gametocidal activity observed by delayed gametocyte maturation and cell deformation (swelling). Computational target prediction indicates the transient receptor potential mucolipin (TRP-ML) transmembrane protein as a potential Calxinin target. Subsequent in vitro validation assays suggest that Calxinin MOA is the modulation of parasite Ca^2+^ levels, as well as that of the host red blood cell (pRBCs). Additionally, the homology modeling of calcium channel conservation among intracellular protozoan parasites shows a <6% homology with human homologs. Validation assays revealed no apparent negative effect on the RBC host T-type Ca^2+^ channels. The validation results of electron microscopy and live cell imaging allow us to construct a hypothesis of Calxinin’s MAO.

## 2. Methods

### 2.1. Synthesis and Purification of Calxinin

2.1.1. (2.R,3S)-3-Amino-4-phenyl-1-(4-(4-(trifluoromethyl)benzyl)piperazin-1-yl)butan-2-ol

Firstly, a dry round-bottom flask was charged with tert-butyl-(S)-1-((R)-oxiran-2-yl)-2-phenylethyl)carbamate 1 (100 mg, 0.38 mol) dissolved in 15 mL of isopropanol, and to this solution 1-(4-(trifluoromethyl)benzyl)piperazine (88.4 mg, 0.45 mol) was added, and the contents were refluxed for 16 h. Thereafter, the reaction mixture was concentrated to remove isopropanol that afforded tert-butyl ((2S,3R)-3-hydroxy-1-phenyl-4-(4-(4-(trifluoromethyl)benzyl)piperazin-1-yl)butan-2-yl)carbamate (compound 2) as a colorless solid in 92% yield, which was used for the next step without further purification. Compound 2 (100 mg) was dissolved in 20% trifluoroacetic acid (TFA) in dichloromethane, which led to the formation of TFA salt in situ, which was further treated with a TFA scavenger basic anion exchange resin (Amberlite IRA-402) to secure a colorless solid product. The obtained solid was purified by column chromatography using neutral alumina gel (1% methanol: chloroform) to isolate Calxinin in an 86% yield. The retardation factor (Rf) was 0.23 in 5% methanol and chloroform.

#### 2.1.2. Tert-butyl-(3-hydroxy-1-phenyl-4-(4-(4-(trifluoromethyl)benzyl)piperazin-1-yl)butan-2-yl)carbamate (2)

Yield: 92%. ^1^H NMR (400 MHz, CDCl_3_): δ 7.53 (d, *J* = 7.8 Hz, 2H), 7.40 (d, *J* = 7.7 Hz, 2H), 7.25 (d, *J* = 3.6 Hz, 4H), 7.17 (d, *J* = 4.0 Hz, 1H), 5.07 (d, *J* = 9.5 Hz, 1H), 3.77-3.61 (m, 3H), 3.50 (s, 2H), 3.00-2.85 (m, 2H), 2.59 (s, 2H), 2.50-2.32 (m, 6H), 2.23 (d, *J* = 10.2 Hz, 1H), 1.38 (s, 9H). ^13^C NMR (100 MHz, CDCl_3_): δ 155.95, 142.55, 138.50, 129.55, 129.23, 128.43, 126.33, 125.25, 125.22, 79.18, 77.57, 77.25, 76.93, 65.58, 62.38, 60.60, 53.38, 53.21, 39.49, 28.44.

#### 2.1.3. 3-Amino-4-phenyl-1-(4-(4-(trifluoromethyl)benzyl)piperazin-1-yl)butan-2-ol (Calxinin)

Yield: 86%. MP. 93-95^o^C. [α]^25.8^_589_ = -30.25 [1% (1g/100 mL) in chloroform]. ^1^H NMR (400 MHz, CDCl_3_): δ 7.55 (d, J = 7.4 Hz, 2H), 7.43 (d, J = 7.4 Hz, 2H), 7.28 (t, J = 6.8 Hz, 2H), 7.20 (s, 2H), 7.19 (s, 1H), 3.60 (d, J = 7.9 Hz, 1H), 3.53 (s, 2H), 2.92—2.83 (m, J = 17.1, 6.5 Hz, 2H), 2.66 (d, J = 7.4 Hz, 2H), 2.64—2.56 (m, 3H), 2.45 (s, 6H), 2.40—2.33 (m, J = 12.3, 2.6 Hz, 1H). ^13^C NMR (100 MHz, CDCl_3_): δ 142.55, 139.20, 129.59, 129.38, 129.27, 128.61, 126.41, 125.69, 125.26, 125.23, 122.99, 77.48, 77.17, 76.85, 68.80, 62.45, 61.20, 55.41, 53.27, 41.47. ^19^F NMR (400 MHz, CDCl_3_): δ–62.5 (s, 3 F). ESI (HR-MS) m/z: (M^+^) calcd for C_22_H_28_F_3_N_3_O 408.2218; found 408.2267.

### 2.2. Maintenance of Reference Pf Parasites and Testing

#### 2.2.1. *Pf* Reference Strains

The laboratory-adapted *Pf* strains such as 3D7 (drug-sensitive) (BEI Resources, MRA-102), D6 MRA-285 (chloroquine (CQ) sensitive, BEI Resources), Dd2 (CQ-resistant, MRA-150, BEI Resources, contributed by David Walliker), and IPC4912 (BEI Resources, NIAID, NIH: *Pf*, strain IPC 4912, MRA-1241, contributed by Didier Ménard.) were acquired from the Malaria Research and Reference Reagent Resource Center (MR4) (Manassas, VA, USA), new BEI resources [18], and maintained with type O-positive erythrocytes suspended in continuous complete culture medium, as described by our team [19,20]. Complete culture media consisted of the Roswell Park Memorial Institute 1640 (RPMI-1640), 4-(2-hydroxyethyl)-1-piperazine-ethanesulfonic acid (HEPES) supplemented with 10% (*v/v*) human serum (H5667, Sigma Aldrich, St. Louis, MO, USA), 92.6 mg/L L-glutamine, 50 mg/L hypoxanthine, and 2 g/L sodium bicarbonate (Sigma-Aldrich, St. Louis, MO, USA). Parasite samples of volume ‘V’, from a liquid nitrogen tank, were incubated at room temperature in a 12% sodium chloride solution (1/5 V) for 5 min before the addition of 9 V 1.6% sodium chloride for 5 min, then 9 V 0.9% sodium chloride for 5 min then centrifuged at 800× *g*. The pellet was washed in a culture medium and then added to 0.25 mL cells and 4.75 mL culture medium. Cultures were incubated at 37 °C and maintained in a low oxygen atmosphere (5% O_2_, 5% CO_2_, and 90% N_2_). The levels of parasitemia in the cultures were maintained between 2 and 10% with 5% hematocrit. The media was changed every 24 h, and parasitemia was monitored every 48 h. Synchronous cultures were prepared by sorbitol treatment [21].

#### 2.2.2. Preparation of Drug Dilutions and Test Plates

Stock solutions of Calxinin and dihydroartemisinin (DHA) were prepared in anhydrous tissue culture grade dimethyl sulfoxide (DMSO; Fisher Scientific, Walthm, MA, USA), while CQ and lumefantrine (LUM) were prepared in sterile distilled water and absolute ethanol respectively, to a stock concentration of 10 mM. The prepared stock solutions were used immediately or stored at −80 °C for later use. Stock solutions were further diluted in serum-free RPMI 1640 media (stock solution solvent final concentration = 0.05%) before performing a 2-fold serial dilution to achieve dose ranges of 0.2 to 50 µM (Calxinin), 0.17 to 87.5 nM (DHA), and 0.1 to 2000 nM (CQ). 25 μL of the drug diluents were aliquoted into 96-well plates and used immediately.

#### 2.2.3. In Vitro Antiparasitic Sensitivity Testing

An SYBR^®^ GREEN I assay procedure with minor modifications was used for drug susceptibility testing of the parasites [22,23]. Briefly, parasite cultures with >1% parasitemia were diluted to 1% parasitemia and 2% hematocrit, and 200 μL was transferred onto drug predosed 96-well plates and incubated at 37 °C for up to 72 h [19,20]. Drug exposure was terminated by freezing the drug plates at −80 °C for 24 h; after which, lysis buffer containing 100 mM Tris-HCl, 10 mM EDTA, 0.016% saponin, 1.6% Triton X-100, and 20× SYBR Green I dye were added, and the samples were incubated for 2 h in the dark. Relative fluorescence units (RFUs) were read using a fluorescence plate reader (SpectraMax^®^ M5 Multi-Mode Microplate Reader, SoftMax^®^ Pro 7.0.2 Microplate Data Acquisition and Analysis Software, Molecular Devices, San Jose, CA, USA), with excitation and emission wavelengths of 485 nm and 535 nm, respectively. For analysis, the readouts were aligned with corresponding drug doses and statistical analyses performed by Prism software (GraphPad, La Jolla, CA, USA). The drug concentrations (x-values) were transformed using the equation X = Log_[X]_ and plotted against the counts (y-values) and the data were analyzed by nonlinear regression (sigmoid dose-response/variable slope equation) to yield the mean 50% growth inhibitory concentration (IC_50_).

#### 2.2.4. Clinical Train Testing

##### Clinical Samples and Study Participants

This study was conducted under the approval of the Kenya Medical Research Institute (KEMRI), Scientific and Ethics Review Unit (SERU), and Walter Reed Army Institute of Research (WRAIR) Institutional Review Boards, protocol numbers WRAIR#2257, KEMRI/SERU/CCR/0018/3126 entitled “Assessment of in vitro antiplasmodial activities of compound libraries for malaria drug discovery and development”. Written informed consent was obtained from a parent/legal guardian, and assent was obtained from individuals aged 13–17 years following the Kenyan law. The fresh field *Pf* parasites used in this study were obtained using a protocol entitled, “Epidemiology of malaria and drug sensitivity patterns in Kenya” under ethical approval of Walter Reed Army Institute of Research Institutional Review Board (study protocol number WRAIR 2454) and KEMRI Scientific and Ethics Review Unit (study protocol number KEMRI 3628). Patients aged six months and above presenting at outpatient departments with symptoms of malaria and/or testing positive for uncomplicated malaria by rapid diagnostic test (mRDT; Parascreen^®^ (Pan/*Pf*), Zephyr Biomedicals, Verna Goa, India) were recruited into the study after providing written informed consent or assent. The clinician then performed the diagnosis by assessing symptoms such as conjunctival pallor, lymphadenopathy, and splenomegaly. The final diagnosis results were based on clinical evaluation confirmed by mRDT and/or microscopy. All *Pf*-positive cases were treated with artemether–lumefantrine (AL) Coartem^®^ as per the Kenya Ministry of Health’s recommended case management guidelines for uncomplicated malaria. The dose of AL was based on weight and the initial dose administration was directly observed and the remaining doses were dispensed to the participant to take at home. Each patient was given the remainder of the full dose of Coartem^®^ and advised to take the next dose after eight hours with a follow-up with the remaining doses at 12 hourly intervals until dose completion. The participants were encouraged to return to the hospital should symptoms persist.

##### Antiplasmodial Activity Testing in Pf Field Strain

*Pf* isolates obtained from natural infections were tested within 6 h, post-phlebotomy, without culture adaptation (immediate ex vivo) for susceptibility to Calxinin and reference antimalarial drugs. Blood samples with greater than 1.0% parasitemia were adjusted to 1.0% parasitemia at 2% hematocrit. Those samples, at less than 1.0% parasitemia, were tested unadjusted at 2% hematocrit. Culture media for the *Pf* field isolates testing was prepared as described Akala et al. 2011 [24] from 10.4 g RPMI 1640 cell culture medium (Invitrogen, Inc., Carlsbad, CA, USA) augmented with 2 g glucose (Sigma Inc., Marlborough, MA, USA) and 5.95 g HEPES (Sigma Inc., Marlborough, MA, USA) dissolved to homogeneity in 1 L of deionized water and sterilized with a 0.2-μm filter. Complete RPMI 1640 media, used for all parasite cultures and drug dilutions, consisted of basic RPMI 1640 media enriched with 10% (*v*/*v*) human ABO pooled plasma, 3.2% (*v*/*v*) sodium bicarbonate (Thermo Fisher Scientific Inc., Waltham, MA, USA), and 4 μg/mL hypoxanthine (Sigma Inc., Marlborough, MA, USA). Complete RPMI 1640 media was stored at 4 °C and used within two weeks. The drugs were assayed using a non-radioisotopic assay technique described by Smilkstein and co-workers in 2014 [25] with modifications by Cheruiyot et al., 2016 [23]. Briefly, the standard reference drug, CQ, and the test drug Calxinin were dissolved in anhydrous DMSO and diluted in complete RPMI 1640 media. Subsequently, two-fold serial dilutions of 0.977 to 2000 ng/mL for both drugs were prepared on a 96-well plate, such that the amount of DMSO was equal to or less than 0.0875%. In vitro drug testing was initiated when the culture-adapted *Pf* at 5% hematocrit with greater than 3% parasitemia was adjusted to 2% hematocrit and 1% parasitemia, then added to the plate containing a dose range of drugs and incubated in a gas mixture comprised of 5% CO_2_, 5% O_2_, and 90% N_2_ at 37 °C. Similarly, for the immediate ex vivo assay, fresh field isolates were centrifuged at 800× *g*, buffy coat removed then washed thrice in basic RPMI 1640 media to remove white blood cells. Parasitemia was estimated by counts based on the number of infected RBCs per 100 RBCs. The pellet containing infected RBCs at 1% was tested without adjusting parasitemia. Pellets containing greater than 1% parasitemia were reconstituted by lowering parasitemia to 1% and hematocrit to 2% in fresh RBCs and tissue culture medium for initiating drug testing assays. Pellets containing less than 1% parasitemia were not tested. Each drug was tested in three biological replicates (in three 96-well plates). The assay was terminated after 72 h. SYBR Green dye in the lysis buffer was kept in dark for 24 h as described [23]. The fluorescence intensity was measured from the bottom of the plate with a GENios Plus plate reader with an excitation wavelength of 485 nm, an emission wavelength of 535 nm, gain set at 60, and a flash count set to 10. Parasite growth inhibition was quantified using Prism Software (version 5.02, GraphPad) as described by Johnson et al., 2007 [26] and presented as IC_50_ ± SD.

#### 2.2.5. Gametocyte Production

Gametocyte cultures of each strain were initiated as previously described [27]. Briefly, on day 0, cultures were synchronized at the ring stage by lysis of the flask pellet with 5 volumes of 5% sorbitol for 10 min at 37 °C. Cultures were then initiated at 0.2% parasitemia and 4% hematocrit in a 10 mL volume. The culture was first incubated in RPMI 1640 (Gibco, UK) supplemented with hypoxanthine (Sigma-Aldrich, Waltham, MA, USA), sodium bicarbonate (Sigma-Aldrich, Waltham, MA, USA), and 1.5% (*w/v*) AlbuMAX II. The cultures were monitored for parasitemia daily with media changes on daily basis with 4% constant hematocrit. With ~8–10% parasitemia (around the 8th day), the volume was doubled and the concentration of AlbuMAX was reduced to 1.0% (*w/v*) with hematocrit at 2%. The cultures were monitored daily for the gross decline of asexual stages and populating gametocytes. The media was changed every other day without adding fresh RBCs. Cultures with >8% gametocytes (around the 12th day) were treated with 50 mM N-acetyl-D-glucosamine (D-GlcNac, Sigma-Aldrich, Waltham, MA, USA) and 50 ng/mL of bistratene A for 3–5 days to remove asexual forms. The percentage of asexual forms and gametocytes were counted on Giemsa-stained smears.

#### 2.2.6. In Vitro Gametocyte Growth Inhibition Assay

Aliquots (100 µL) of cultures containing gametocytes at the indicated stages (preferably stage I) were transferred to a 96-well plate in duplicate as described previously [27]. The plate was incubated in a 37 °C incubator maintained with 5% CO_2_. The culture medium was changed after 48 h and the appropriate concentration of Calxinin was maintained. Blood smears of parasites were fixed with methanol and stained with Giemsa (Amresco, Cleveland, OH, USA). Slides were observed and parasitemia was monitored with a light microscope equipped with a 100× apochromatic objective lens. The images were captured by a 10-megapixel digital microscope camera for enumerating the efficacy of Calxinin.

#### 2.2.7. Calxinin Resistant Mutant Selection

Following previously published methods [28,29], we cultured *Pf*3D7 strain (4–5% parasitemia) with fresh media containing 10µg/mL of Calxinin for 48 h and recovered the parasites by changing to fresh media. This process continued until parasitemia reached 4–5% along with the addition of a higher concentration of drug compound (10-µg/mL increments).

### 2.3. Testing the Efficacy of Calxinin in Pb Mouse/Mosquito Malaria Models

#### 2.3.1. In Vitro Model

Mosquitoes were reared under standard conditions (i.e., 26 °C, 80% room humidity, 12 h light-dark cycle, 5% sucrose solution provided through cotton pads). Salivary glands of *Anopheles stephensi* mosquitoes (IDL, NII, India) infected with GFP expressing -*Pb*ANKA (BEI Resources, Manassas, VA, USA) parasites (Insectary at National Institute of Immunology, New Delhi, India) were dissected (infectivity was determined by counting number of sporozoites per mosquito in a hemocytometer) and Sporozoites (IDL, NII, India) were extricated in complete medium (Dulbecco’s Modified Eagle’s Medium (DMEM) containing 10% fetal bovine serum (FBS) and 3x penicillin/streptomycin (P/S), PS stock 100×, Gibco, UK). 20,000 sporozoites/well were overlayed on a monolayer of cultured HepG2 cells (ATCC, USA, Manassas, VI, USA) to allow infection. HepG2 confluency consistency was determined by MTT assay as per the manufacturer’s protocol (Sigma Aldrich Cat. Id. 11465007001). The cells were pretreated with Calxinin at various concentrations (0.1, 1, and 10 µM). The plate was centrifuged at 3000 rpm for 5 min to ensure the sporozoites settled down onto the cells, then placed in an incubator at 37 °C for 3 h. The medium was replaced with a pre-warmed fresh medium (containing DMEM, 10% FBS, 3× P/S, and 0.1% Fungizone) and supplemented with either DMSO or Calxinin at indicated concentrations. After two days, the cells were treated with Trizol and frozen at −80 °C. RNA isolation followed by RT-PCR was performed to estimate the parasite load in the infected cells.

#### 2.3.2. In Vivo Models

##### Erythrocytic Stage

To evaluate the anti-plasmodial activity of Calxinin on the murine model of malaria, we used C57BL6 mice (weight = 25 ± 2 g) and developed malaria infection through intraperitoneal injection of 1 × 10^7^ *Pb* infected erythrocytes. Experiments were performed following the standard protocols approved (Aproval No. NII-IAEC#448/17) by the Institutional Animal Ethics Committee (IAEC) of the National Institute of Immunology, New Delhi (Dr. Agam P Singh’s lab), under the Committee for Control and Supervision of Experiments on Animals (CPCSEA), Government of India. The animals used were 6 to 8 weeks old and housed under standard controlled conditions at 25 °C with a 12-h light-dark cycle. Animals had ad-libitum access to a standard laboratory pellet diet and water. At 48 h post-infection, one group (5 mice) was administered with a single dosage of Calxinin (50 mg/kg, dissolved in 10% DMSO). Another group (5 mice) received vehicle control (10% DMSO without drug) through a subcutaneous route. The onset of infection and efficacy of Calxinin was monitored by measuring the parasitemia using methanol fixed blood smear Giemsa (10%) staining microscopy with blood from the tail vein on days 3, 7, and 10 post-infection. Additionally, we also observed the efficacy of Calxinin on the infected mice survival (mean survival time, MST) with respect to untreated controls until 30 days post-infection. The reduction in parasites was taken as an index of the therapeutic activities of the test compound [30]. The percentage of parasitemia was calculated manually with Cell Counting Aid software, following the formula (total number of parasitized RBCs)/(total number of RBCs) × 100.

##### Liver Stage Infection in Mice, Calxinin Treatment, and Survival Assay

*Plasmodium berghei ANKA* parasites (BEI Resources, USA) were maintained in C57BL/6 mice for a maximum of four serial passages and passed through *Anopheles stephensi* mosquitoes. (IDL, NII, INDIA) GFP-expressing *Pb*ANKA was grown in *Anopheles stephensi* mosquitoes in the insectary at the National Institute of Immunology (New Delhi, India). Sporozoite count per salivary gland and infectivity was determined before the experimental infection of mice through mosquito bites. Briefly, anesthetized mice were exposed to a cage of sporozoite carrying starved mosquitoes (~100 mosquitos/5 mice) for 15 min with intermittent disturbing of the mosquitos (every 2–3 min) to encourage biting and further injection of more sporozoites. Biting was allowed at 22 ± 1 °C and in a dark condition maintained in an incubator. The first Calxinin dose (10 mg/kg, intraperitoneal) was given 24 h before the infection. Subsequent doses were given at 2 and 24 h after the sporozoite challenge (median time of biting session). To calculate the parasite load, the liver of each experimental mouse was isolated 50 h post-infection. Mice were anesthetized, the abdominal area sterilized with 70% ethanol, and the liver was dissected. The excised liver was homogenized in a pre-cooled denaturing solution. RNAs were isolated from the homogenized sample followed by RT-PCR.

#### 2.3.3. Ex Vivo Models

##### The Effect of Calxinin in an In Vitro Ookinete Inhibition Assay

To determine whether Calxinin can block the growth of the transmission stage of malaria, Pb-GFPcon, which expresses the GFP during all the developmental stages, was used in the in vitro experiments. Calxinin was tested at nanomolar concentrations for its ability to inhibit ookinete development. Gametocytes were obtained by pretreating mice with phenylhydrazine and subsequently infecting them with Pb-GFPcon parasites. On day three post-infection, gametocyte-rich blood was harvested. Gametocyte stages were maintained in vitro by incubation in ookinete media containing xanthurenic acid and various other nutrients including complete DMEM (pH 8.0) and incubated at 19 °C for a maximum of 24 h. Ookinete development was followed by Giemsa-stained smears. For the inhibition assay, culture plates containing various Calxinin concentrations were incubated for ~24 h at 19 °C on a shaker in the dark. After 24 h, ookinete development was monitored by making blood smears from the culture. Smears were fixed, stained with Giemsa, and counted using a NIKON 80i microscope.

### 2.4. Human Host Cross-Reactivity

#### 2.4.1. Calxinin Cytotoxicity Testing in Human Cells

A cell cytotoxicity assay was conducted against human peripheral blood mononuclear cells (PBMCs, Loyola University Medical CenterBlood Bank, Maywood, IL, USA), human epithelial kidney (HEK293 (ATCC, USA, Manassas, VI, USA)) cells, and liver cell lines (Huh 7.1 and HEPG2(ATCC, USA)). To assess the toxicity, HEK293 and Huh 7.1 cells, plated in 96-well (1 × 10^3^ cells/well) plates were exposed to varying concentrations of Calxinin for 24 h along with 10/100 µL Alamar Blue solution (10× = 4.8 mM in phosphate buffer) per well [31]. Following incubation, cell viability was measured using the SpectraMax^®^ M5 Multi-Mode Microplate Reader, using a 530 nm excitation wavelength and a 590 nm emission wavelength, to quantify metabolically reduced resazurin dye. Results from the cytotoxicity assays were expressed as CC_50_, defined as the drug concentration at which 50% of the cells died or metabolically slowed down. The average minima and maxima were added for normalizations with the negative control and blanked, respectively, to retain their relative global trend. Briefly, PBMCs were isolated from healthy donors using the Ficoll-Paque^TM^ technique (GE Healthcare Bioscience AB, Helsinki, Finland) from de-identified donor leukopack samples obtained from the Loyola University Medical Center (LUMC, Chicago, IL, USA) blood bank in accord with a contractual agreement. PBMCs were maintained in RPMI 1640 medium supplemented with 10% FBS, and P/S (1% *v/v*) (Gibco, UK) at 37 °C. Robust cells, at a concentration of 8 × 10^4^ cells/mL, were plated into 96-well plates and incubated for 24 h. Six concentrations of Calxinin were added in a two-fold dilution from a starting concentration, ranging from 50 to 1.56 µM, in triplicate and incubated with the cells for 24 h as indicated above.

The cell viability ratio was calculated by the following formula: % cytotoxicity = [Mean optical density (OD) of test cells]—[Mean OD of control cells]/[Mean OD of control cells] × 100. Viability counts were then plotted against corresponding drug concentrations to yield cytotoxicity (CC_50_) through nonlinear regression (sigmoid dose-response/variable slope equation). Drug CC_50_ values were then used to define a selectivity index (SI), which is a measure of drug safety in humans relative to parasitic cells, and was calculated as the ratio of the CC_50_ value, determined on the PBMC/HEK293 cells (cytotoxicity), to the IC_50_ value, determined on parasite growth inhibition *Pf* 3D7/Dd2 (anti-plasmodial activity). In this study, we set a SI as the cut-off to indicate low drug cytotoxicity.

#### 2.4.2. Patch-Clamp (T-Type)

The piperazine substructure of Calxinin was known to block T-type calcium channels in humans [32]. Though there was no significant binding predicted to negate host cross-reactivity, The α1H cDNA in the vector pcDNA3 was used to transfect, 1 × 10^6^ HEK 293 cells (ATCC, Manassas, VI, USA) were transfected with 10 μg of α1H plasmid using Lipofectamine (GIBCO-BRL, Gaithersburg, MD, USA), according to the manufacturer’s protocol [33], were plated in 6-mm round coverslips precoated with poly-L-lysine (1 h at 37 °C) and incubated in a 5% CO_2_ humidified atmosphere at 37 °C in high glucose minimum essential medium supplemented with 10% FBS, 1% glutamine, 1% P/S, and 600 mg/mL G-418 sulfate (geneticin, Cellgro, Herndon, VA, USA). Currents were recorded with the patch-clamp technique in the whole-cell configuration using an Axopatch 200B amplifier, pClamp 10 software, and a Digidata 1440 digital-analog converter (Molecular Devices, San Jose, CA, USA). Data was acquired with a 2-kHz filter and digitized at 20 kHz. The pipette filling solution consisted of (in mM) 108 CsMeSo_3_, 4 MgCl_2_, 1 Cs-EGTA, 9 HEPES, and 5 ATP-Mg Tris (pH 7.4; 280 mOsmol/kg H_2_O). The external solution consisted of: 5 CaCl_2_, 140 TEACl, and 10 HEPES (pH 7.4, 290 mOsmol/kg H_2_O). Pipettes were produced from borosilicate glass (Sutter Instruments, Novato, CA, USA) with resistances between 3 and 7 MΩ. Only cells with series resistance (Rs) <10 MΩ were used. The Rs were compensated online (>80%). The currents were developed from a maintenance potential (V_h_) equal to −90 mV, depolarizing the membrane in test pulses of 300 ms from Ȓ90 mV to +40 mV in 20-mV increments (Supplementary Figure S2).

#### 2.4.3. Evaluation of the Hemostatic Effects of Calxinin

*Plasmatic blood clotting assays.* Calxinin was diluted to prepare a stock concentration of 200 µg/mL and further diluted in citrated normal human pooled plasma over a concentration range of 2.5–20 µg/mL. Calxinin-supplemented plasma samples were assayed to determine prothrombin time (PT; Innovin, Dade, Miami, FL, USA), activated partial thromboplastin time (aPTT; Platelin, Trinity Biotech; Bray, Co., Wicklow, Ireland), and thrombin time (bovine thrombin; Enzyme Research Laboratories, South Bend, IN). All assays were performed on an ACL 3000 fast kinetic coagulation analyzer (Beckman-Coulter, Brea, CA, USA). *Thromboelastography.* This assay was performed on freshly collected whole blood samples using a TEG 5000 system (Haemonetics, Boston, MA, USA). 304 μL citrated blood and 36 μL of the test compound were added to each sample cup. Some (20 μL of 0.02 M) calcium chloride was added to recalcify the sample and initiate clotting. The clot tracing was monitored for 30 min. The system software calculated parameters that characterized the clot formation. A compound’s effect on the initiation and amplification phases of blood coagulation was characterized in terms of its effects on R time and K time. The effects on clot strength were quantitated in terms of the maximum amplitude of the tracing. The speed of fibrin production and cross-linking were characterized by the angle parameter. *Platelet aggregometry.* Citrated whole blood was differentially centrifuged to produce platelet-rich and platelet-poor plasmas. Platelet-rich plasma and Calxinin (final concentration = 20 µg/mL) were added to aggregation cuvettes and incubated at 37 °C. After 2 min, a platelet agonist was introduced to the cuvette and platelet aggregation was monitored for 10 min using a PAP-8 platelet aggregometer (BioData, Horsham, PA, USA). The platelet agonists tested included: ADP (5 µM), epinephrine (500 µg/mL), collagen (10 µg/mL), and thrombin receptor activating peptide (10 µM).

### 2.5. Calxinin Target Prediction In Silico

#### 2.5.1. Target Search

The 3D models of Calxinin binding were developed using the steepest descent algorithm with force field UFF, where all atoms move in Avogadro software [34]. Calxinin was analyzed by a battery of servers in search of possible targets like TargetHunter, Pharmmapper, Spider, SuperPred, Stitch, Hitpick, reversescreen3D, and Swiss target prediction [35,36,37,38]. Both Stitch and Swiss target servers suggested voltage-gated calcium channels as top possible hits. The basis of these consensus predictions was reported with the binding of highly pharmaco-similar compounds to calcium channels [32,39].

There is little information regarding the identity of calcium channels in the *Plasmodium* proteome let alone their crystal structures. Therefore, sequence similarity and data mining were performed using pore and calcium-sensing domain S4 homology within the *Plasmodium* proteome [40] and KEGG orthologous mapping [41] of all of the calcium-permeable channels identified in unicellular protozoans [42]. All the leads were subjected to a homology search using the plasmoDB database BLAST tool [40]. Out of a total of 35 homologs, 6 (PF3D7_0212500; 1313500; 1362300; 0719900; 0106300; 1211900) (VEuPathDB Bioinformatics Resource Center, San Francisco, CA, USA) were shortlisted based on essentiality established by piggyBac mutational survival assay [43,44]. The 3D models of all 6 proteins were predicted by the I-Tasser server [45,46,47] and refined by the FGMD tool [45]. The primary amino acid sequence of all 6 proteins was retrieved from the PlasmoDB server [40]. Further, the sequences were subjected to pore homology and IPRscan motif/domain mapping [48,49]. The large proteins (~1700 amino acids) were truncated to 1500 amino acid lengths, which covered most of the functional domains. Active regions were predicted by the COFACTOR server [50]. The final protein models were subjected to molecular docking with Calxinin by Patchdock-Firedock [32,39].

The piperazine moiety containing flunarizine and lomerizine backbones of Calxinin interacted with a cation/voltage sensing S4 transmembrane helix having positively charged arginine/lysine amino acid residues that act as a switch, keeping the channel closed when the extracellular space has fewer cations or less voltage. To compare this with the human voltage-gated channels, T-type (PDB id: 4mw8) and N-type (PDB id: 5gjw) were docked with Calxinin and previously reported compounds by rigid docking (Patchdock), followed by Firedock for flexible refinements [32,39]. While the reported compounds NP118809/NP078585 against N-type [39], Z941/Z944 against T-type [32], and compound IDs ‘14′ and ‘17′ against TRPV6 calcium channels [51] showed interactions with the positively charged domain, interestingly, no satisfactory interaction with the host channel pore was predicted for Calxinin.

#### 2.5.2. Phylogenetic Analysis of the Putative Calxinin Target

The nucleotide sequences of PF3D7_1313500 of isolates from different parts of the world were obtained from PlasmoDB [40]. The sequences were translated into amino acid sequences using the ExPASy translate tool and were aligned using the ClustalΩ server [52]. Multiple sequence alignments (MSA) were performed with homologs from different *Plasmodium* species [53] and with other apicomplexan/kinetoplastid parasites and the Calxinin binding region was mapped. For multiple sequence alignments, orthologous genes of PF3D7_1313500 were retrieved from the database of KEGG [54] and EuPathDB [55] and subjected to ClustalΩ protein sequence alignment [52]. The PF3D7_1313500 orthologs of *Plasmodium* species used were *P. vivax* (PVX_122495), *P. knowlesi* (PKNH_1414100), *Pf* (PF3D7_1313500 and PfIT_13001880), *Pb* (PBANKA_1411900), *P. chabaudi* (PCHAS_1413800), *P. malariae* (*PmUG01_1414100*) and *P. ovale* (PocGH01) (VEuPathDB Bioinformatics Resource Center, USA). Phylogenetic analysis was carried out for retrieved sequences using the online server phylogeny.fr (http://www.phylogeny.fr/phylogeny.cgi (accessed on 21 April 2021)) [56] by submitting ClustalΩ output files. Briefly, a phylogenetic dendrogram of all homologous sequences within Plasmodium species was constructed using the ‘phylogeny.fr’ server and employing a Neighbor-joining method and bootstrap proportions of 1000 replications. Phylogenetic relationships with PF3D7_1313500 orthologous genes of other parasites closely related to Plasmodia (i.e., *Cryptosporidium* spp., *Toxoplasma gondii, Trypanosoma* spp., *and Leishmania* spp.) (VEuPathDB Bioinformatics Resource Center, USA). were also processed similarly. Further, to evaluate the negative, positive, or neutral impact of single or multiple amino acid substitutions on the Calxinin interaction, the binding was assessed by mapping the interaction regions on the MSA. The figures were made by processing alignment outputs by the Boxshade server [57].

#### 2.5.3. In Silico Validations of the Putative Calxinin Target

The target complex with Calxinin was subjected to MD simulation and trajectory analysis conducted using the Desmond module of Schrodinger software [58]. The lipid bilayer was constructed using membrane placement methodology within the maestro environment (POPC phospholipid models) selecting membrane parsing domains predicted by the PPM server [59]. The full system was prepared using Maestro’s Protein Preparation Wizard. The system was solvated in TIP3P water models with 0.15 M NaCl and placed in solution. A simulation box covering the whole system was placed with a 10 Å buffer space. The simulations were run for 100 ns at 300 °K and standard pressure (1.01325 bar) at a constant surface tension (NPγT). The OPLS-AA 2005 force field parameters were elected for utilization for both this preparation and all later simulations.

### 2.6. Target Confirmatory Assays

#### 2.6.1. Confocal Live Imaging

Two tubes with 0.3 mL (3% hematocrit) of 4–5% parasitemia culture of *Pf*3d7 cells with mixed stages of parasites were washed and identically loaded with Fluo-4 AM (Invitrogen™ Molecular Probes, Inc, Eugene, OR, USA). The loading buffer contained a modified Hank’s buffered salt solution (HBSS) with Ca^2+^ and magnesium (Mg^2+^). Next, the loading buffer, along with HBSS, containing 0.1 mg/mL bovine serum albumin and 10 mM probenecid was adjusted to pH 7.4 with sodium hydroxide. Cells were loaded with Fluo-4 AM (5 μM) for 30 min at 37 °C in the dark with intermittent resuspension [60]. The parasites were washed with a loading buffer without Fluo-4 AM, transferred to glass-bottom culture plates (Mattek Life Sciences, Ashland, MA, USA) precoated with poly-L-lysine, and incubated for 15 min. The cells were again washed with the loading buffer and then transferred to an LSM 510 confocal microscope equipped with an Axio Observer Z1 motorized inverted microscope, incubator, heating unit, heated stage, temperature controller, and humidifier (Carl Zeiss Microscopy). For the duration of the imaging, the parasites were maintained at 37 °C and 95% CO_2_/5% O_2_. Fluo-4 AM was excited with the 488 nm line of the Argon/2 laser and emissions between 505 and 550 nm were measured. The samples were induced with different concentrations of CQ (control) or Calxinin in parallel with corresponding vehicle controls. RBCs were dynamically imaged using differential interference contrast (DIC) microscopy.

#### 2.6.2. Super-Resolution Live Imaging

Live Airyscan imaging was performed in Super Resolution mode on an LSM 880 Airyscan microscope (Carl Zeiss Microscopy, Jena, Germany) equipped with a 63 × 1.4 numerical aperture objective (Carl Zeiss Microscopy, Jena, Germany). The sample processing was identical to the above. Raw data were processed using Airyscan processing in “auto strength” mode (mean strength ± SD = 5.5 ± 1.3) with Zen Black software (version 2.3, Carl Zeiss Microscopy, Jena, Germany).

#### 2.6.3. Electron Microscopic Evaluation of Calxinin’s Effect on the Subcellular Localization of Pf Ca^2+^

3-3′-Diaminobenzidine (DAB) photoconversion transmission electron microscopy (TEM) experiments were performed in accord with a published method [61] with minor modifications to determine the effects of Calxinin on the subcellular localization of Ca^2+^ in *Pf* within infected RBCs. Two 0.3 mL (3% hematocrit) of 4–5% parasitemia culture of *Pf*3d7 cells in RBCs with mixed stages of parasites were washed and identically loaded with Fluo-4 AM (Invitrogen™ Molecular Probes, Inc., Eugene, OR, USA). The loading buffer was modified HBSS with Ca^2+^ and Mg^2+^. The loading buffer, along with HBSS, contained 0.1 mg/mL bovine serum albumin and 10 mM probenecid and was adjusted to pH 7.4 with sodium hydroxide. Cells were loaded with Fluo-4 AM (5 μM) for 30 min at 37 °C in the dark with intermittent resuspension [60]. After the first wash with Fluo-4 AM freeloading buffer, the samples were treated with either DMSO or 500 nM Calxinin for 10 min. After induction, the samples were washed thrice with Ca^2+^ and Mg^2+^-free Dulbecco’s phosphate-buffered saline solution (DPBS), then resuspended in phosphate-buffered saline (PBS) (Thermo Fisher Scientific, Waltham, MA, USA) containing 2% paraformaldehyde and 1% glutaraldehyde (Electron Microscopy Sciences, Hatfield, PA, USA) and left at room temperature for 3 h. Thereafter, the cells were washed twice with PBS and incubated for 3 h under a germicidal UV lamp (UV-C) with the DAB solution replaced every half an hour with a fresh solution of DAB. The DAB solution was freshly prepared by dissolving 20 mg of DAB (Millipore Sigma, St. Louis, MO, USA) in 1 mL DMSO, briefly vortexed, then brought to 30 mL with PBS. Fixed and photoconverted cells were washed thrice with distilled water and post-fixed with distilled water containing 2% osmium tetroxide (Electron Microscopy Sciences, Hatfield, PA, USA) and 1.5% potassium ferricyanide (Electron Microscopy Sciences, Hatfield, PA, USA) for 1 h at room temperature. The cells were rinsed several times with distilled water, stained en bloc with uranyl acetate (Electron Microscopy Sciences, Hatfield, PA, USA), dehydrated by incubation in an ascending series of alcohols (25, 50, 75, 95, and 100%, Electron Microscopy Sciences, Hatfield, PA, USA) followed by incubation in 1:1 propylene oxide: epoxy resin (comprised of a mixture of EMbed 812, nadic methyl anhydride (NMA), dodecenyl succinic anhydride, and 2,4,6-Tris- (diethylaminomethyl)phenol, Electron Microscopy Sciences, Hatfield, PA, USA) for 12 h at room temperature on a rotary mixer (Ted Pella, Inc., Redding, CA, USA). Next, the cells were incubated with 100% epoxy resin for 12 h at room temperature on a rotary mixer (Ted Pella, Inc., Redding, CA, USA). The epoxy resin was changed, and the cells were incubated for 2 h at room temperature on a rotary mixer (Ted Pella, Inc., Redding, CA, USA) and then were baked at 70 °C for 72 h. Ultrathin sections (gray, 50 nm) were cut with an ultramicrotome (EM UC7, Leica Microsystems, Vienna, Austria), mounted on formvar- and carbon-coated 400 mesh grids (Electron Microscopy Sciences, Hatfield, PA, USA), stained with uranyl acetate and lead citrate, and visualized with a Philips CM 120 transmission electron microscope (TSS Microscopy) equipped with a BioSprint 16-megapixel digital camera (Advanced Microscopy Techniques, Woburn, MA, USA).

### 2.7. Data Analyses

All the in vitro screening of infected cells was performed in triplicate [62]. Parasite inhibition data were analyzed by nonlinear regression to yield the IC_50_ (Prism software, Graph Pad), as previously described [19,26,27]. CC_50_ values were derived similarly to antiparasitic IC_50_ values by plotting transformed drug concentrations, using the formula X = Log _[X]_, against cell viability counts (ODs) to yield the CC_50._ An SI value (i.e., the ratio between a drug’s cytotoxic CC_50_ and parasitic IC_50_ value) ≥ 2.0 (~20-fold) was used as the cut-off for low cytotoxicity to PBMCs. After Calxinin established a low cytotoxicity, according to the aforementioned criteria, it was advanced to high-throughput screening with the IC_50_ cut-off value set at <2 µM [29,63]. Comparisons between treatment groups were performed by ANOVA and Dunnett’s post hoc comparisons. For the in vitro assays of hemostatic compatibility, concentration–response curves were developed for each of the lead compounds using 3–5 plasma or whole-blood samples. These concentration–response curves were analyzed by two-way ANOVA followed by Holm-Sidak multiple comparison tests.

### 2.8. Image Analysis

Images were acquired with Zen software (Carl Zeiss Microscopy) and analyzed offline with Imaris Software (version 9.5, Bitplane, Zürich, Switzerland). The number and fluorescence intensity of Fluo-4 positive (Fluo-4+) *Pf* infected RBCs (iRBC) were assessed by using the spots function of Imaris image analysis software (version 9.5, Bitplane, Zürich, Switzerland). Fluo-4 spots were semi-automatically detected and classified as positive or negative using an XY diameter equal to 4 µm and with background correction. The fluorescence intensity of the Flou-4+ spots was measured as the intensity of the voxel at the center of the spot. All images were analyzed using identical image analysis parameters. Data were exported to Prism software (GraphPad) for statistical analysis. Differences between the control and Calxinin-treated cells were assessed using unpaired *t*-tests. The number of Fluo-4+ spots over time was analyzed via nonlinear regression. An α level of 0.05 was used for statistical tests.

### 2.9. Statistical Analysis

All experiments were performed in triplicate or more as stated. Statistical analyses were performed using GraphPad Prism (GraphPad Software, La Jolla, CA, USA). For each experiment, means ±standard deviations (SD) were calculated. Statistically significant differences were identified using Student’s *t*-test, with *p*-values < 0.05 considered statistically significant.

## 3. Results

### 3.1. Rational Antimalarial Design and Synthesis

**Figure 1:**
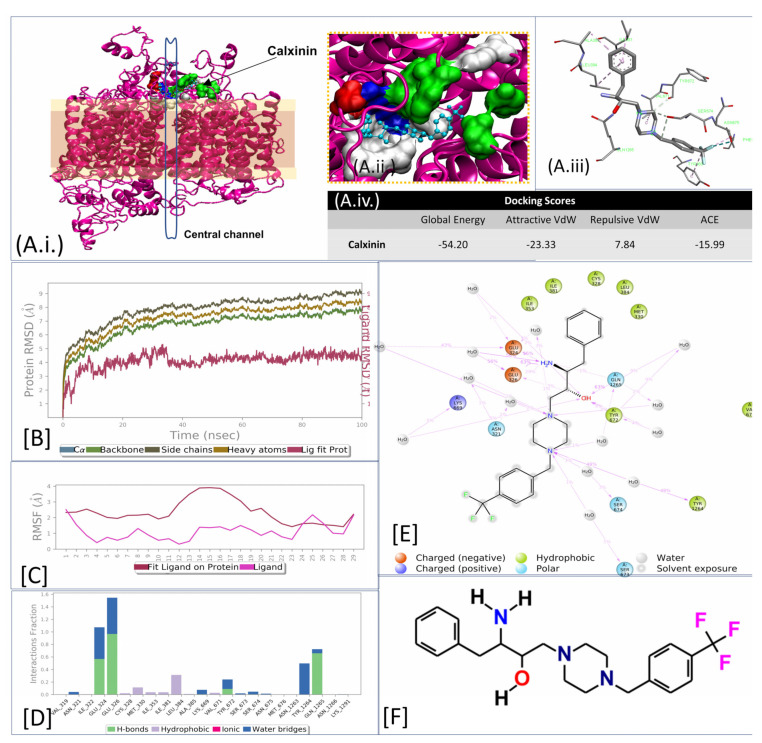
(**A**) Schematic representations of Calxinin docked at the active site (**i**) Active site amino acid molecular 3D topological positions and relative position of the central channel pore. Color and representation scheme: The protein backbone is mauve and in ribbon cartoon. (**ii**) Zoom-in view of binding pocket with docked ligands. Calxinin binding site is represented by amino acid side chain residues in surface structure colored by type (white: hydrophobic, green: polar, red: -ve, and blue: +ve charged), (**i**,**ii**). Calxinin is represented by CPK in sky blue color. Dashed lines show the blown-up frame of the docking site. (**iii**) Licorice styled wireframe model to show 3D interacting side-chain amino acid residues and interaction type. (**iv**) Initial docking scores table with global energy, attractive and repulsive Vanderwall forces, and atomic contact energy (ACE). (**B**) A 100 ns MD simulation data analysis for Calxinin. RMSD of the protein backbone/ligand position relative to the initial structure throughout 100 ns MD simulations. (**C**) Ligand vs. binding site movements during the simulation. (**D**) Graphical representation of the percentage of protein-ligand contacted and their biochemical nature throughout the MD simulation. (**E**) Two-dimensional interaction map and interaction biochemical characteristics most conserved during the simulation. The phenyl trifluoride group seems not to interact due to changes in its binding site during simulations lowering the percent interaction of the interacting pocket. (**F**) Two-dimensional structure of Calxinin, ((2S,3S)-3-amino-4-phenyl-1-(4-(4-(trifluoromethyl)benzyl)piperazin-1-yl)butan-2-ol).

In continuation of our previous work on antimalarial agents, we functionalized benzyl piperazine with the trifluoromethyl group to study the effect of this substitution on the antimalarial activity of the compound. The use of organofluorinated molecules offers valuable avenues for the design of new potential drug candidates against malaria. The incorporation of fluorine substituents, particularly trifluoromethyl groups, into organic molecules has led to their high potency against many diseases, including malaria. The noteworthy characteristics of fluorine include high electron affinity, lipophilicity, and bioavailability, extending the half-life of the drugs [16,64]. These modifications may help to increase the lipophilicity of the molecules and further enhance their activity. This newly designed HEA-based piperazine compound (Calxinin) resulted from our lead optimization process (Figure 1F). We implemented the epoxide ring-opening using 1-(4-(trifluoromethyl)benzyl)piperazine as described in the literature [65]. This resulted in the desired compound, Calxinin at an 86% yield. The composition of Calxinin was confirmed by nuclear magnetic resonance (NMR) (^1^H, ^13^C, and ^19^F) spectroscopic techniques and high-resolution mass spectrometry (HR-MS) (Data not shown). To the best of our knowledge, this is the first report of the combination of a ((trifluoromethyl)benzyl)piperazine with HEA. 

### 3.2. Plasmodium Falciparum Antiparasitic Activity Breakpoints

#### 3.2.1. Blood Stage Antimalarial Activity of Calxinin in the Reference Strain

The initial assessment of the antimalarial activity of Calxinin on asynchronous cultures of the chloroquine-sensitive *Pf*3D7 strain, using an SYBR Green assay, showed highly potent antiparasitic activity with a mean IC_50_ value of 88.0 (±1.1) nM. Calxinin activities against various *Plasmodium* strainsm and different developmental stages are summarized in Table 1.

#### 3.2.2. Effect of Calxinin on the ART-Resistant Field Strain IPC 4912

Calxinin showed activity against the artemisinin-resistant *Pf* strain IPC 4912 from BEI Resources with an IC_50_ of 93.0 nM (Table 1), which is comparable to the concentrations at which other strains are inhibited. This data suggests that the activity of Calxinin is independent of the mechanism of resistance by artemisinins.

#### 3.2.3. Calxinin Activity in *Pf* Field Isolates from a Malaria-Endemic Region

Following confirmation of Calxinin’s activities against lab strains in vitro, blood stages of the mouse, liver stages, and mosquito stages, we tested its efficacy against freshly collected field isolates (in parallel with reference clones). *Pf* field isolates were obtained from natural infections, without prior culture adaptation [24]. Rich within-infection clonal diversity [66] and high genetic diversity in the Kenyan parasite population have also been reported in these isolates [67] The drug sensitivity profiles and the evaluation of genetic markers for drug resistance in field isolates have been described previously [68,69]. The results showed that Calxinin shows antimalarial activity with mean IC_50_ values below 100 nM, confirming the high potency against the field strains. The documentation of compounds with good to excellent activity against these isolates represents field potency. This is in contrast to the activity against laboratory reference strains, such as PfNF54, and clones, such as Pf3D7, that underwent controlled series of laboratory culture cycles for more than 20 years [66]. For example, using 24 replicates of in vitro assays, *Pf* parasites showed IC_50_s of 135.0 ± 6.7 nM (*n* = 8), which is higher than the IC50 for freshly collected field isolates.

#### 3.2.4. Activity of Calxinin against Gametocytes

**Figure 2:**
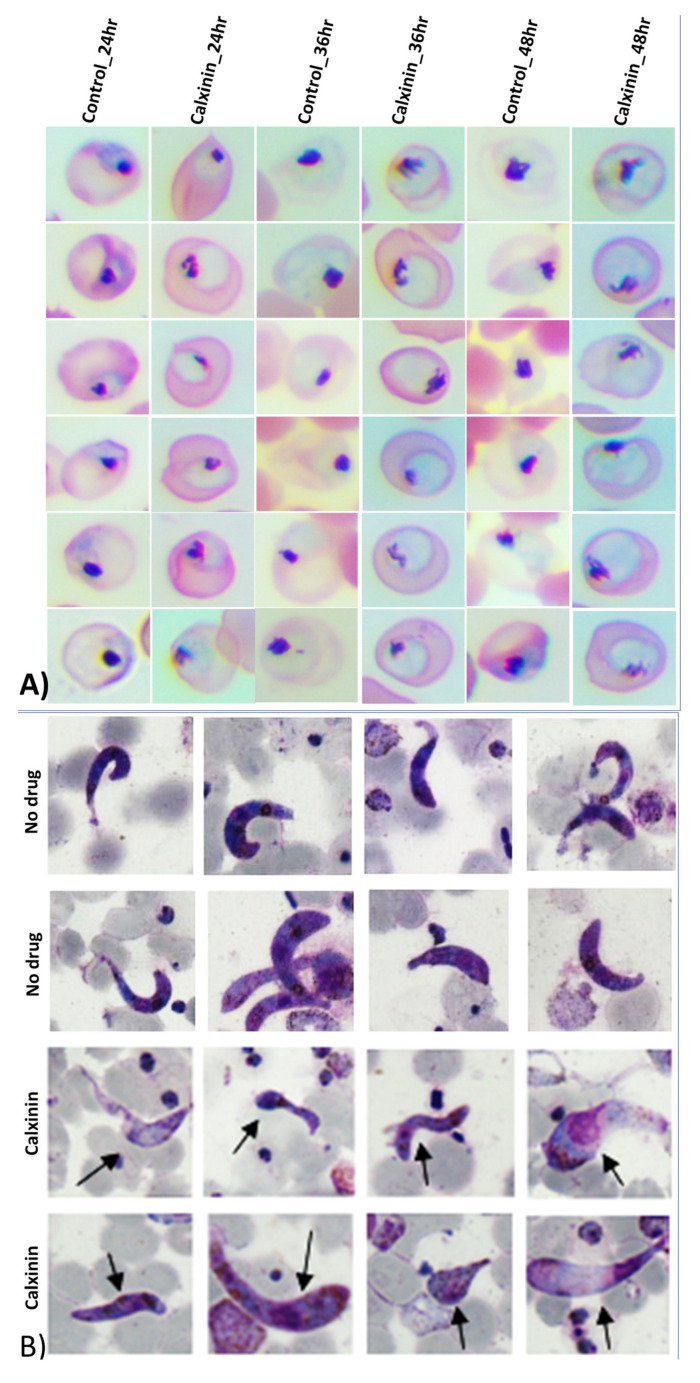
Effect of Calxinin on sexual stage and mosquito stage parasites. (**A**) A 100-µL 3rd, 4th, and 5th-day gametocyte-rich cultures were seeded in a 96-well microtiter plate in triplicates for each treatment (0.5 µM). Untreated control samples in triplicate were fixed and Giemsa stained. Morphological characterization revealed a reduction in the maturation of remaining viable gametocytes in Calxinin treated wells and made them appear more flaccid and circular, specifically evident as mature forms start appearing in control groups (48 h, far right vertical panel). Calxinin has a detrimental effect on the normal morphology to an extent that none of the treated samples had any mature free gametocytes. (**B**) Structural changes in the ookinete formation are observed at indicated concentrations of Calxinin. The top two horizontal panels are representative images of the control (healthy) ookinete. The bottom two horizontal panels are representative images of the deformed and retarded Ookinete resulting from the treatment with Calxinin (450 nm). Images were acquired with a NIKON 80i microscope at 100× magnification. Smears were fixed with methanol and stained with Giemsa.

Gametocytes were grown and exposed to varying concentrations of Calxinin (starting from 0.5 µM) as described in the Methods. This exposure resulted in a decreased maturation of stage III–V gametocytes (*Pf* 3D7, 41.0 ± 3.1% inhibition) and morphologically distorted parasites when compared to the DMSO-treated control (Figure 2A). Calxinin’s effect on *Pf* gametocyte viability and morphology was evident as gametocytes often appeared viable but were unfit. Gametocyte morphology was monitored for 72 h and changes were compared to DMSO-treated control gametocytes. Gametocytes receiving Calxinin exhibited a swollen appearance, which resulted in the absence of the typical crescent shape (Figure 2A). This shows Calxinin’s potential as a gametocidal agent. It also supports our hypothesis of Ca^2+^ dysregulation as an underlying mechanism for Calxinin as Ca^2+^ is essential for maintaining parasite morphology. Figure 2A shows images of gametocytes fixed at different time points. Gametocytes pictured at 72 h and compared to 48 h or 24 h, respectively, show Calxinin’s fast and lasting activity. DMSO alone did not have any effect on gametocyte development or morphology.

### 3.3. Calxinin Antiparasitic Activity in the Plasmodium berghei Mouse Model Breakpoints

#### 3.3.1. Efficacy of Single-Dose Calxinin on In Vivo Blood-Stage *P. berghei* in Mice

**Figure 3:**
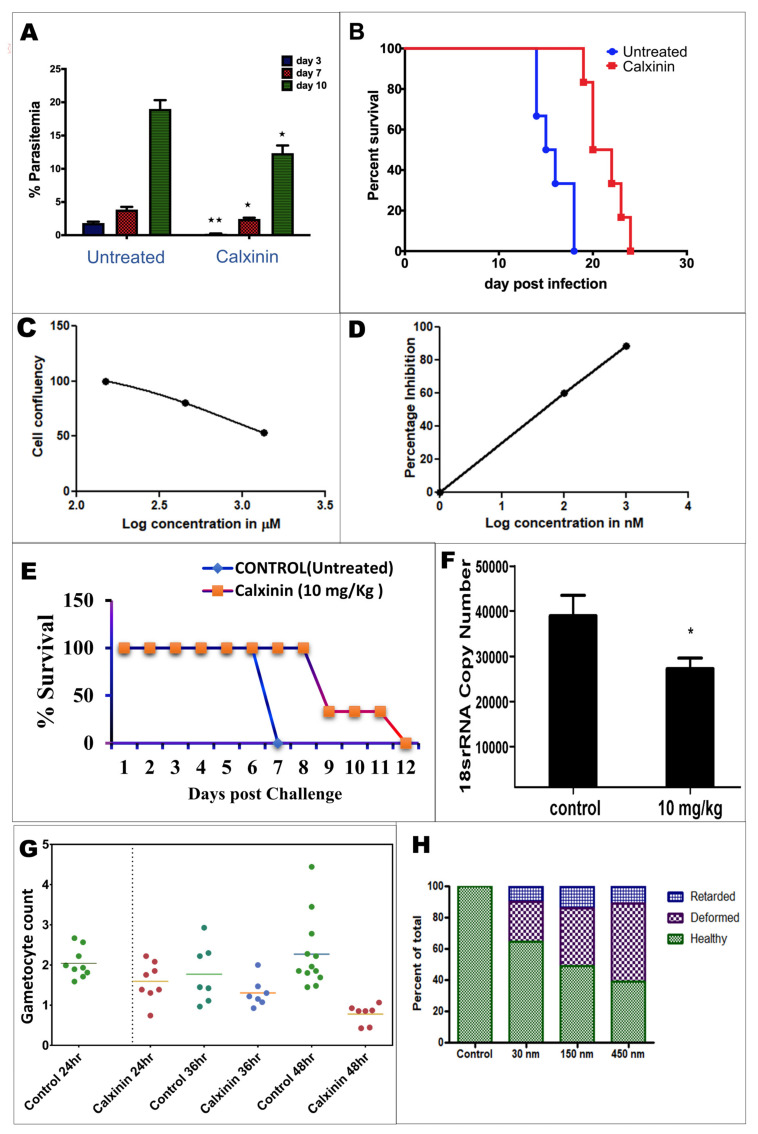
Effect of Calxinin on blood-stage parasites and survival of *P. berghei* NK65 infected mice. (**A**) The animals were treated with a single dose of Calxinin (50mg/kg) and monitored for parasites using Giemsa-stained blood smears. The data represent the M ± SD of 5 animals in each group. For the in vivo experiments, statistical differences between the two groups were determined by Student’s *t-*test. (**B**) The mean survival time (MST) of the mice was followed up to day 30 post-infection using Kaplan-Meier survival analysis, and the statistical difference in animal survival was analyzed by a log-rank test. (**C**) In vitro cytotoxic effect of Calxinin on HepG2 cells was carried out before infecting with *P. berghei* and cell viability is determined using MTT assay. (**D**) HepG2 cells were infected with *P. berghei* sporozoites and treated with Calxinin at the indicated concentration. % inhibition is plotted. Calxinin is active against the liver-stage infection of *P. berghei* in culture. (**E**) Mice were challenged with sporozoites through infected mosquito bites. *P. berghei* sporozoite load per mosquito gland was 11,000. On day 0, day 1, and day 2, mice were treated with Calxinin at 10 mg/kg (i.p). Survival was monitored until all the mice died. Mean survival times (MST) between control and Calxinin were 6 days and 9 days, respectively (*n* = 4 per group). (**F**) The efficacy of 10 mg/kg Calxinin against *P. berghei* liver stage infection in a mouse model; here, readings are mean ± SD of the 18SrRNA copy number from the experiments of triplicate samples plotted using tabular results of *t*-test (*p =* 0.0165). (**G**) Treatment with Calxinin for 24, 36, and 48 h decreased gametocyte counts by ~50% in 48 h and a 100% reduction in mature gametocytes. Each scattered plot column represents different sets of samples from parasites treated at different time points (*p_value_* < 0.05) compared to the control. (**H**) In vitro ookinete inhibition assay against PbGFPcon. * significant i.e., *p* < 0.05, ** highly significant i.e., *p* < 0.01.

We observed that treatment of *P. berghei* infected mice with a single subcutaneous dose of Calxinin (50 mg/kg) on day 2 post-infection significantly reduced the parasite load. Parasitemias of treated mice were 0.21% on day 3, 2.45% on day 7, and 12.3% on day 10. In comparison, the untreated control group exhibited a parasitemia of >19% by day 10 (Figure 3A). The mean survival time of the Calxinin-treated group versus the vehicle-treated group was 21 days and 15.5 days, respectively (Figure 3B), indicating that Calxinin treatment significantly increased the survival time. Notably, a single dose of Calxinin did not completely cure the mice of infection as recrudescence was evident after day 10 post-infection. The observed effect could be due to the limited pharmacokinetics and stability of Calxinin in the blood circulation. Yet, our results indicate that even a single dose of Calxinin as a promising antimalarial effects in an experimental Pb mouse in vivo model.

#### 3.3.2. Activity of Calxinin against Parasite Liver Stages In Vitro and In Vivo

After the injection of sporozoites by mosquitoes into the human bloodstream, the liver schizonts are the first stage of human infection. The activity of candidate drug compounds against these liver stages is considered an important part of antimalarial therapy known as chemoprotection. To evaluate the efficacy of Calxinin against liver-stage parasites, we infected cultured HepG2 cells with *Pb* sporozoites previously harvested from mosquito salivary glands. Infected HepG2 cells were treated with Calxinin (0.1, 1, and 10 μM) dissolved in 1% DMSO. Subsequently, the parasite load was quantified by qPCR in the control and Calxinin-treated cells using parasite-specific 18S rRNA primers. Approximately 60% and 88% reduction of the parasite load was observed in cells treated with 0.1 µM and 1 µM of Calxinin, respectively. IC_50_ values were determined to be 79 nM (Figure 3C,D). We treated the infected mice with a fixed dose of 10 mg/kg of Calxinin for three days. *Pb* liver-stage parasites matured into liver schizonts within ~ 55 h. The parasite load was quantified by qPCR and we observed 30 % inhibition at a given dose of 10 mg/kg Calxinin (Figure 3F). Notably, the liver stage potency of Calxinin was much higher than that of atovaquone (ATQ) in the culture [70]. Next, we investigated the liver stage activity of Calxinin in the mouse model by infecting mice via mosquito bites). We recorded a single-day delay in the survival of Calxinin-treated mice over the untreated controls. The gain in survival time was notably less (Figure 3E) when compared to the gain observed for the blood stage infection (Figure 3B). This may be because infection with sporozoites leads to a dramatic parasite amplification event in liver cells.

#### 3.3.3. *Plasmodium berghei* Ex Vivo Model Antiparasitic Activity Breakpoints; In Vitro Ookinete Inhibition Assay for Calxinin

**Figure 4:**
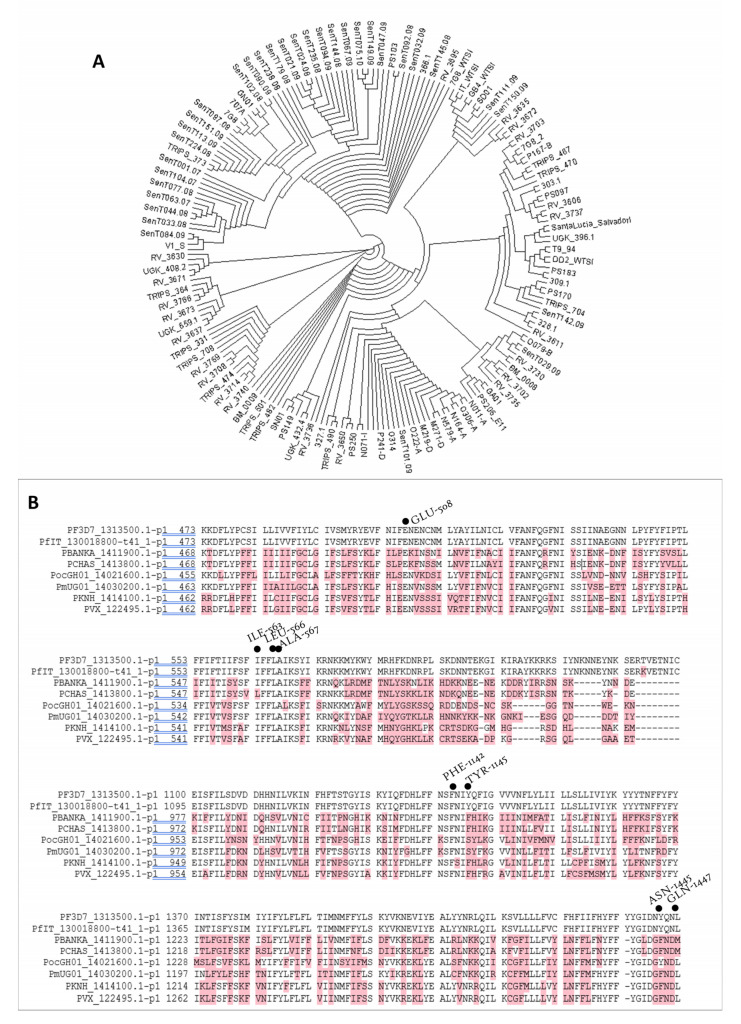
Phylogenetic and sequence analysis of the Calxinin putative target. (**A**) Phylogenetic tree of PF3D7_1313500 sequence homologs generated using data mined 218 translated genome sequences of *P. falciparum* field isolates from around the world. All sequences formed phylogenetic groups according to evolutionary niches due to geographical location or time of isolation. This shows high conservation of the gene product among these isolates. Calxinin key interacting residues include: GLU-508, ILE-563, LEU-566, ALA-567, PHE-1142, TYR-1145, ASN-1445, and GLN-1447. These residues were 100% conserved in all 218 isolates. (**B**) Multiple sequence alignment (MSA) segments of PF3D7_1313500 homologs from different *Plasmodium* species. The binding pocket highlighted by a ‘black pointer’ was highly conserved biochemically among all species, highlighting its conservation even among distant species. This analysis shows low chances of spontaneous mutations and thus a low likelihood of developing resistance due to functional importance. Any change in the biochemical nature of the pocket will have a high fitness cost for the parasite.

Encouraged by the notable potency of Calxinin against asexual blood stage, liver stage, and sexual stage parasites, we evaluated the transmission-blocking efficacy of the compound. Calxinin showed remarkable inhibition against *Pb* ookinete development (IC_50,_ 150 nM) in culture (Figure 4B). As the concentration of the Calxinin increased, the percentage of healthy parasites was significantly reduced, while the percentage of deformed ookinetes increased. Microscopic images of the healthy, deformed, and retarded ookinetes affected by Calxinin are shown in Figure 4B. The effect is strikingly similar to its effect on *Pf* gametocytes (Figure 4A) and further supports our hypothesis of calcium homeostasis disturbances as the main MOA of Calxinin. The deformed and retarded parasites most likely cannot continue their development, thus being unable to form oocysts. Overall, Calxinin shows high potency to inhibit ookinete development and eventually oocyst formation, suggesting its capability to block malaria transmission.

#### 3.3.4. Resistant Variant Selection

Parasite cultures (*Pf*3D7) were treated with incremental concentrations of Calxinin for 48 h. The recovery time following 10-µg/mL and 20-µg/mL exposure were 7 and 10 days, respectively. Parasites treated with 30 µg/mL were rendered nonviable and, hence, unrecoverable. Our experiment failed to select resistant isolates as the cultures frequently crashed at even low Calxinin concentrations, resulting in ‘sluggish’ growth rates. Parasite cultures that were subjected to multiple cycles of exposure showed no change in IC50 for Calxinin, dihydroartemisinin (DHA), or chloroquine (CQ). The Calxinin target in plasmodium may have a high functional cost for viable mutants selection under culture conditions. To identify the potential target(s) of Calxinin in *Plasmodium,* we opted for a computational approach by using an in silico pipeline to predict target(s) and elucidate the mechanism of action of Calxinin.

#### 3.3.5. In Silico Target Elucidation

Using the publicly available PlasmDB [REF] with a focus on the *P. falciparum* 3D7 proteome our pipeline identified a putative *P*. *falciparum* membrane protein, such as a channel or a pump, as a potential Calxinin target. Among the six modeled essential and putative channels/pumps/transporters in the *Plasmodium* database (i.e., PF3D7_0212500, 1313500, 1362300, 0719900, 0106300, and 1211900), the proteins that exhibited the most significant pore homology with T-type and N-type channels of humans were PF3D7_0212500 (32.03% pore homology) and 1313500 (19.28% pore homology). The amino acid residues of all constructed models were within the allowed regions of the Ramachandran plot; (>90%). The C- score of all models was < −3.00, the expected TM Score was <0.5, RMSD values were within 11–15, and z-scores were less than -5.00. The docking scores for Calxinin were highest within the PF3D7_1313500 pore region (Figure 1A(iv)). PF3D7_1313500 is a putative transient receptor potential cation channel of the mucolipin subfamily (TRP-ML), which may be involved in the Ca^2+^ homeostasis of the parasite.

#### 3.3.6. PF3D7_1313500 Genetic Polymorphism

The Calxinin key interacting residues were 100% conserved in 218 genomes sequenced *Pf* isolates in the plasmoDB database (Figure 4A). Sequence alignments with homologous proteins from other intracellular parasites showed high conservation of the Calxinin interacting residues (Figure 4B).

#### 3.3.7. Target Validations through the In Silico Approach

Molecular Docking (MD) simulations for 100 nanoseconds were performed for the 2R,3S enantiomer (Calxinin) top docking poses with the PF3D7_1313500 pore region (Figure 1B–D). Calxinin showed a highly stable complex-forming tendency throughout the 100-nanosecond simulation. Binding with Calxinin caused restrictions in movements of interacting chains shared by the pore region of the channel likely affecting pore dynamics of opening and closing.

#### 3.3.8. Calxinin’s Effect on *Pf* Intracellular Ca^2+^ Levels

**Figure 5:**
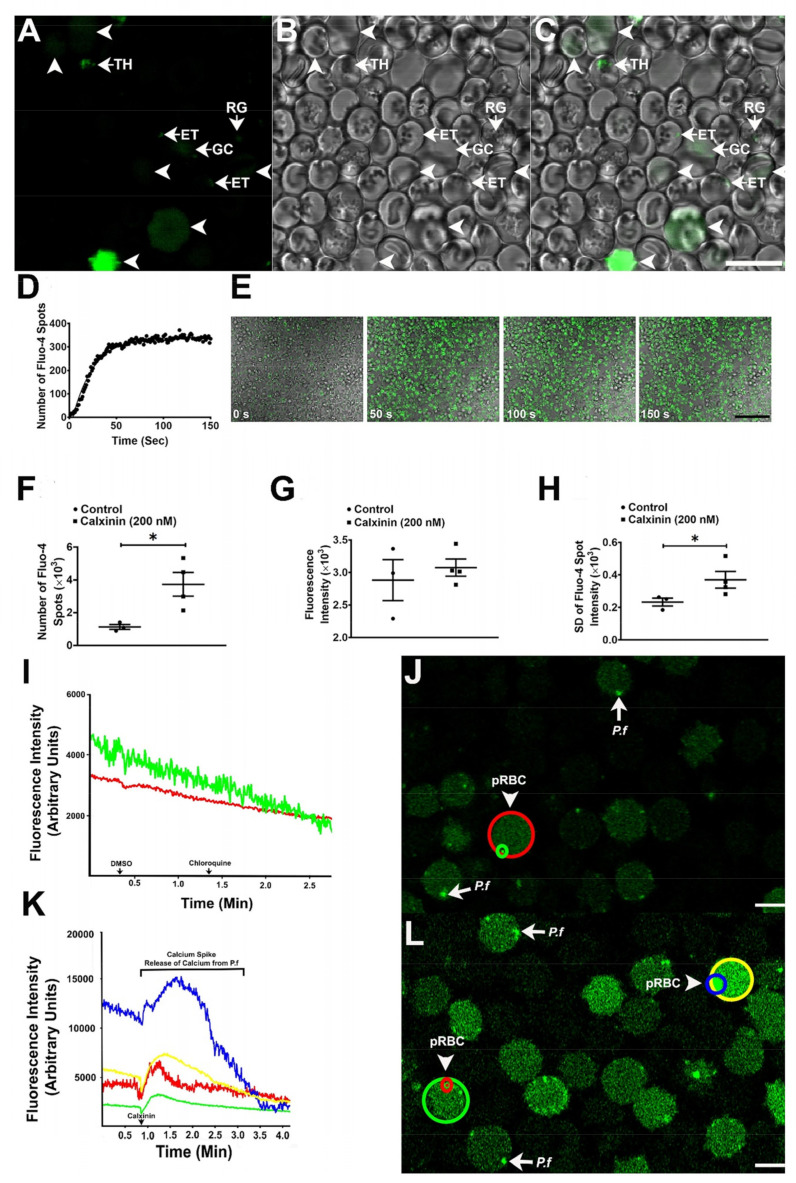
Confocal and live imaging of Fluo-4 AM-loaded uninfected and infected RBCs (2 to 3% parasitemia). (**A**) Fluo-4 AM fluorescence in uninfected and infected RBCs. (**B**) Visualization of RBCs by DIC microscopy. (**C**) Merged Fluo-4 fluorescence and DIC microscopy. The white arrowheads point to older, uninfected RBCs that have a higher Fluo-4 fluorescence than younger RBCs due to their older in vivo age. The white arrows point to parasites within RBCs at different stages. Abbreviations: ET, early trophozoite stage; TH, trophozoite stage; RG, ring stage; GC, gametocyte. The scale bar in (**C**) is equal to 10 µm and is valid for A-C. Treatment of *P. falciparum*-infected RBCs with Calxinin (200 nM) increased the intracellular Ca^2+^ concentration of *Pf* and/or the RBCs. (**D**) Live confocal imaging revealed an exponential increase in the number of Fluo-4+ *Pf*-infected RBCs after the addition of Calxinin to the culture media that plateaued at ~60 s. (**E**) Representative merged Fluo-4+ (green) and negative *Pf*-infected RBCs (visualized by DIC microscopy) at 0, 50, 100, and 150 s after the addition of Calxinin to the culture media. (**F**) Treatment of *Pf*-infected RBCs with Calxinin increased the number of Fluo-4+ RBCs. (**G**) Treatment of *Pf*-infected RBCs with Calxinin did not affect the fluorescence intensity of the Fluo-4+ RBCs. (**H**) Treatment of *Pf*-infected RBCs with Calxinin increased the SD of the fluorescence intensity of the Fluo-4+ RBCs. The data in (**E**–**H**) is given as *M* ± *SEM* (*n* = 3 control; *n* = 4 Calxinin-treated). The scale bar given in E equals 50 µm and is valid for all panels. * *p* < 0.05, unpaired *t*-test. (**I**) Ca^2+^ quantification in *Pf*-infected RBCs. (**I**). Parasitized RBCs (pRBC, 5%) were treated with either chloroquine (CQ, 10 µM) or DMSO. The arrows in (**I**) indicate the time that DMSO and CQ were added to the culture media. The red line in (**I**) is the fluorescence intensity over time in the pRBC circumscribed with a red circle in (**J**). The green line in (**I**) is the fluorescence intensity over time in the parasite within the RBC circumscribed with a green circle in (**J**). No changes in Ca^2+^ were observed in DMSO- or CQ-treated RBCs or parasites. Experiments were performed in triplicate. (**J**) A representative image of pRBCs after DMSO and CQ was added to the culture media. The fluorescence intensity was quantified in pRBCs (red circle) and *Pf* within the RBC (green circle). White arrowheads point to pRBCs. White arrows point to *Pf* fluorescence within pRBCs. The scale bar equals 5 µm. (**K**) The treatment of parasitized RBCs with DMSO did not affect the Ca^2+^ levels of pRBCs and parasites, and the treatment with Calxinin (10 µM) produced a robust increase in the Ca^2+^ levels (hereafter referred to as a Ca^2+^ spike) of pRBCs and parasites within 0.1 s. DSMO was added to the culture media at a time equal to 0 s; Calxinin was added to the culture media at the time indicated by the arrow in (**K**). The yellow line in (**K**) is the Ca^2+^ spike observed after Calxinin treatment in the pRBC circumscribed with a yellow circle in (**L**). The blue line in (**K**) is the Ca^2+^ spike observed in the parasite within the RBC circumscribed with a blue circle in (**L**). The green line in (**K**) is the Ca^2+^ spike observed after Calxinin treatment in the pRBC circumscribed with a green circle in (**L**). The red line in (**K**) is the Ca^2+^ spike observed in the parasite within the pRBC circumscribed with a red circle in (**L**). Experiments were performed in triplicate. (**L)** A representative image of pRBCs after DMSO and Calxinin were added to the culture media. The fluorescence intensity was quantified in pRBCs (yellow and green circles) and *Pf* within the RBC (blue and red circles). White arrowheads point to pRBCs. White arrows point to *Pf* fluorescence within pRBCs. The scale bar equals 5 µm.

To determine if Calxinin affects intracellular Ca^2+^ concentration of *Pf* and/or the infected RBCs, either Calxinin (200 nM, final concentration) or an equivalent volume of DMSO was added to the culture media and the *Pf-*infected RBCs. The culture was imaged once per second for 2 min. Fluo-4 AM is a cell-permeant dye that is used to measure intracellular Ca^2+^ concentration (Figure 5A–L). Fluo-4 AM is non-fluorescent until bound to Ca^2+^. The hydrophobic AM moiety allows its passage across the cell membrane of viable cells. Once inside, intracellular esterases cleave the AM groups, leaving Fluo-4 trapped inside the cells and free to bind with intracellular Ca^2+^. The degree of Fluo-4 fluorescence increases greater than 100-fold after it is bound to Ca^2+^ [71] (Figure 5E). The addition of Calxinin to the culture media resulted in an exponential increase in intracellular Ca^2+^ concentration that reached a plateau at ~60 s (Figure 5D). In contrast, the addition of DMSO or CQ to control cultures at a higher concentration than Calxini) did not affect the intracellular Ca^2+^ concentration (data not shown). Given that the Calxinin-induced increase in intracellular Ca^2+^ concentration reached a plateau at ~60 s, subsequent live cell images were acquired 60 s after the addition of Calxinin. In these experiments, Calxinin treatment increased fluorescence in *Pf* -infected RBCs by greater than three-fold and decreased the variability in the intensity (Figure 5D–H).

#### 3.3.9. Determination of Intracellular *Pf* Ca^2+^ Levels through DAB Photoconversion TEM

**Figure 6:**
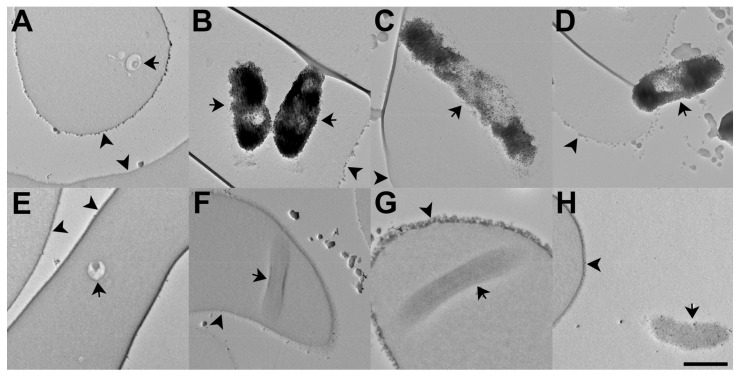
Transmission electron micrographs of pRBCs treated with Calxinin. Treatment of 500 nM Calxinin leads to a profound decrease in intracellular Ca^2+^ concentration in *Pf* trophozoites, schizonts, and ring stage. (**A**–**D**) Representative electron micrographs of DMSO-treated *Pf* in the ring, early trophozoite, late trophozoite, and schizont stages, respectively. Arrowheads point to the RBC plasma membrane. Arrows point to *Pf*. (**E**–**H**) Representative electron micrographs of Calxinin-treated *Pf* in the ring, early trophozoite, late trophozoite, and schizont stages, respectively. Arrowheads point to the RBC plasma membrane. Arrows point to *Pf.* The intracellular Ca^2+^ concentration is profoundly lower in the ring, early and late trophozoite, and schizont stage *Pf* after the treatment with Calxinin compared to DMSO-treated pRBCs (ring stage: compare arrow in (**A**) to that in (**E**); early trophozoite stage: compare arrows in (**B**) to that in (**F**); late trophozoite stage: compare arrow in (**C**) to that in (**G**); schizont stage: compare arrow in (**C**) to that in (**G**)). The scale bar in E equals 400 nm and is valid for (**A**,**E**). The scale bar in H equals 600 nm and is valid for (**B**–**D**) and (**F**–**H**).

To visualize the subcellular localization of Ca^2+^ and determine the effect of Calxinin on intracellular Ca^2+^concentration, *Pf* -infected RBCs were loaded with Fluo-4 AM and then treated with either 500 nM Calxinin or DMSO for 10 min. The Fluo-4 AM was photoconverted to an electron-dense reaction product by incubation with DAB in the presence of ultraviolet light. Importantly, the Ca^2+^ concentration is directly related to the amount and intensity of the electron-dense reaction product. Next, the cells were prepared for imaging by TEM. We observed that Calxinin (500 nM) treatment produced a significant decrease in intracellular Ca^2+^ concentration in parasites in the ring, early and late trophozoite, and schizont stages. In contrast, neither DMSO or Calxinin (500 nM) affected the intracellular Ca^2+^ concentration of uninfected RBCs (Figure 6).

#### 3.3.10. Measuring Ca^2+^ Levels through Fluo4-AM Staining and Super-Resolution Live Microscopy

Encouraged by the results in the previous experiments, we used Fluo-4 AM to determine the effect of Calxinin on the regulation of Ca^2+^ in the parasite and the host RBC. In these experiments, images were recorded at an acquisition rate of 80 frames per second. Infected RBCs and parasites were identified via DIC Images and were subsequently recorded in the “confocal” configuration. The dye was excited with the 488 nm line of the Argon laser, and its emission was measured at 550 nm. Under these conditions, parasites can be readily identified as they show much stronger Ca^2+^ signals than RBCs. Consequently, infected RBCs exhibit a stronger Ca^2+^ signal than uninfected RBCs. After measuring the baseline, Calxinin was added to the medium at a concentration of 200 nM and the emission of Fluo 4 was continuously recorded. The results of a typical experiment are shown in Figure 5J,L, in which we observed the changes in Fluo-4 fluorescence as a consequence of the internal Ca^2+^ fluctuations. Interestingly, approximately 50–90 s after adding Calxinin (Figure 5K), a two-phase increase in the parasite’s internal Ca^2+^ concentration occurs. In the first and faster phase, a maximum Ca^2+^ concentration is reached in approximately 80 s and then begins to decrease. A second phase immediately follows showing a lower Ca^2+^ concentration with an increased rate and a higher maximum value. This is followed by a decrease in parasite internal Ca^2+^ levels, reaching a fluorescence level that is lower than the initial baseline. This increase in RBC Ca^2+^ concentration follows the increase in the parasite’s Ca^2+^ concentrations. In control cells, the addition of DMSO or CQ did not affect the Ca^2+^ concentration of pRBCs (Figure 5I,J). Likewise, no change in fluorescence was observed in nonparasitized RBCs. The negative control data are not highlighted here due to the observation of a much lower Ca^2+^ level and, hence, much lower fluorescence in the nonparasitized RBCs.

### 3.4. Host Toxicity and Host Calcium Homeostasis Interference

#### 3.4.1. Cytotoxicity Effects of Calxinin on Human Primary and Cell Lines

The cytotoxicity of Calxinin was evaluated using human primary cells, as well as established human cell lines. PBMCs (peripheral blood mononuclear cells), HEK293 cells, and liver (Huh 7.1 and HepG2) cells were exposed to various Calxinin concentrations. Exposure of HEK293, Huh7.1, and HepG2 cells to Calxinin did not result in any toxicity. Interestingly, we observed that Calxinin seemingly enhanced the growth of PBMC (% PBMC viability >100% relative to the vehicle control) at the same mean IC_50_ value that is required to kill the parasite. To determine the CC_50_ values, we treated the cells with various concentrations of Calxinin and titrated them at five-fold serial dilution. In these dose-response assays with PBMCs, the Calxinin treatment showed only mild cytotoxic effects even at high concentrations. The CC_50_ was 1000 μM. Similarly, the Calxinin treatment had no cytotoxic effect on the human cell lines. The CC_50_ values were as follows: 60.2 μM for HEK293 cells, 100.0 μM for Huh 7.1 cells, and 1,166.0 μM for HepG2 cells.

#### 3.4.2. Effect of Calxinin on Human Calcium Channel

Although the sequence homology data from the computational analysis indicated that Calxinin most likely would have no affinity for human T-type calcium channels, we performed experiments to determine whether Calxinin cross-reacts with human calcium channels. HEK-293 cells stably expressing voltage T-type Ca^2+^ channels (α_1H_; Cav3.1) were subjected to various Calxinin concentrations, ranging from 200 nM to 1 µM (Note that 1 µM is >10 times Calxinin’s IC_50_ value against erythrocytic stages). There was a 20% reduction in amplitude with Sipatrigine (positive control) at 10 µM (IC50~30 µM data not shown). Either Calxinin or DMSO (vehicular/negative) was added directly into the experimental chamber after seal formation and basal currents were recorded. Supplementary Figure S2 shows that the inward currents became measurable at −60 mV (V_h_ = −90 mV) and reached a peak at −50 mV, with a peak current density of –48.05 ± 2.23 pA/p.f. As shown, the addition of Calxinin at any concentration did not produce significant changes in the dependence of potential or in the maximum peak current density, which was −46.22 ± 3.89 pA/p.f for 1 µM Calxinin (*n* = 6; *p* > 0.05).

#### 3.4.3. Effects of Calxinin on Blood Hemostatic Parameters

The impact of Calxinin on hemostatic parameters in human blood was assessed following supplementation to plasma and whole blood collected from healthy individuals. Clotting assays to assess the extrinsic pathway (prothrombin time; PT), intrinsic pathway (activated partial thromboplastin time; aPTT), and common pathway (thrombin time; TT) of blood coagulation were performed following supplementation with Calxinin at concentrations of up to 20 µg/mL. Our results revealed that clotting times in the presence of Calxinin were not different from vehicle-supplemented samples (Supplementary Figure S3A–C). In vivo blood clot formation is dependent not only on plasmatic clotting enzymes but also on the circulating blood cells, particularly platelets. To test this, freshly drawn whole blood was supplemented with Calxinin (20 µg/mL) or vehicle, and the clot formation was assessed using thromboelastography. In these experiments, Calxinin did not affect the clot formation profile (Supplementary Figure S3D). It also did not alter platelet responsiveness in an agonist-induced aggregation assay (Supplementary Figure S3E,F). Overall, these data suggest that Calxinin does not have any effect on the normal homeostasis of human blood cells.

## 4. Discussion

Combating drug resistance requires the continued development of new antimalarials that target multiple essential metabolic pathways and, ideally, several developmental stages of the parasite [3]. Existing therapeutics mostly target the disease-causing blood stages of the parasite. Limited successful efforts have been made to target other developmental stages, such as the liver stages, gametocytes, and mosquito stages. Therefore, drug development strategies should ideally include novel compound (s) that possess activity against multiple stages of the malaria parasite. It should also be effective against drug-resistant strains. Furthermore, compounds should exhibit favorable homeostatic properties and minimal or no toxicity to the host. Incorporating such compounds into existing combination therapies may reduce the selection of resistant strains [72,73,74,75]. Here, we present a novel compound called ‘Calxinin’ named after its underlying MOA in releasing Ca^2+^ from parasites in infected red blood cells. The compound was developed from the HEA and piperazine backbone [13,14,19,20]. Piperazine analogs with medicinal value were previously used to build several potent antimalarial agents [13,14,19,20,27,76]. The biological activities of HEA analogs have been extensively studied for their antimalarial activity by our team [13,14,19,20,27] and others [77,78,79]. For instance, the HEA analog KAF156 is currently in Phase 2 clinical trials for treating malaria [6]. For the development of Calxinin, additional functional groups were added to the piperazine core to improve bioactivity. Notably, HEA-based compounds with known bioactivity have already entered early clinical trials for their anti-Alzheimer’s disease activity [13,80,81].

Conventional antimalarial treatments are being developed primarily on their antiplasmodial activity against parasites in cell culture and often with an incomplete understanding of the molecular mechanism of action [3]. Recently, parasite protein targets and validation of drug mechanism of action have been facilitated by computational approaches taking advantage of protein 3D structure modeling and Molecular Docking simulations. These approaches guide drug discovery efforts in a cost-saving and time-efficient manner [82,83]. Molecular docking is a computational procedure that attempts to predict the non-covalent interactions of drug molecules (ligands) to larger macromolecules (target) [84,85]. The technique results in multiple leads that require experimental validation [86,87,88].

Our in silico analysis and MD simulations indicated an uncharacterized putative cation channel of the malaria parasite (TRP-ML) as a potential target for Calxinin. We have based our predictions on SAR reports of similar calcium channel blockers [32,89]. The compound binding site stability and favorable interactions could only be assessed reliably through MD simulations [90] as the target pool is comprised mainly of uncharacterized proteins. The reported mutagenesis data indicate that the protein is essential in the parasite [40,44]. The sequence is highly conserved among sequences from diverse *Pf* isolates from different geographical regions of the world. In particular, C-terminal amino acid side chain residues that we predict to be critical for predicted Calxinin binding are 100% conserved. Orthologs in several pathogenic parasite genera show high conservation of the predicted Calxinin-binding residues, suggesting a fundamental significance in terms of function and possibly regulation in apicomplexan and kinetoplastid parasites. These in silico predictions were the basis of examining the calcium homeostasis in parasites. The results from live imaging and electron microscopy experiments strongly indicate calcium homeostasis in *Pf* is affected by Calxinin. Calxinin does not seem to have any direct effect on homologous proteins in the human host. This is expected, as none of the putative calcium channel/pump proteins shares significant homology with the host mammalian homologs Ca^2+^ storage in human cells differs from that in parasites (absent calcisomes). Yet, Ca^2+^ signaling and homeostasis are just as critical for the *Pf* life cycle. This includes key steps in host cell invasion, egress from host cells, and genomic regulation [39,91]. Furthermore, Ca^2+^ regulates apicoplast metabolism, which plays a key role in parasite growth [92]. Specific experiments are required to completely unravel and exploit the Calxinin/target interaction to generate a repertoire of novel inhibitors specific to calcium channels/pumps/transporters, as they are known to be vulnerable to a range of small molecule inhibitors/modulators [53]. The MD simulations pointing to molecular interference with the calcium-sensing domain of this channel open new avenues for exploring this new type of channel interaction. Crystallographic and structural studies will be required to determine the precise channel modulation changes that are induced by calxinin.

Experimentally supporting our computational predictions are results that show the alteration of the Ca^2+^ homeostasis of *Plasmodium*-infected RBCs by Calxinin. Increased intracellular calcium alters the cytoskeleton and causes cell injury by facilitating swelling and/or affecting membrane permeability [93]. The swollen/flaccid appearance of gametocytes and ookinetes in the live imaging and electron microscopic data also supports our hypothesis that Calxinin affects calcium homeostasis in the parasite. The putative target protein is expressed in all developmental stages and is crucial for parasite survival. Additionally, Calxinin’s multistage activities may significantly broaden its application not only as an antimalarial therapeutic agent but also as a prophylactic and/or transmission-blocking agent. We demonstrate the potent antimalarial activity of Calxinin against CQ- and ART-resistant laboratory strains of *Pf*. The ART-resistant profile did not affect the parasiticidal breakpoints of Calxinin. This could mitigate the evolution of drug resistance as calxinin acts against novel target proteins [94]. Calxinin also exhibits potent activity against clinical *Pf* strains from malaria-endemic regions of Kenya. This demonstrates that the activity of the compound is not restricted to lab-optimized *Plasmodium* strains and would be useful in the field without further major optimization.

Calxinin is active against parasites in the malaria mouse model, which is often used as the first in vivo test of potential antimalarials but also to specifically investigate the malaria mosquito stages. Our data demonstrate that Calxinin also effectively blocks malaria transmission in the mosquito. These results would expand the use of Calxinin for chemoprophylaxis.

An evaluation of the cytotoxicity of Calxinin was conducted in human primary cells, as well as in lab-established human cell lines. It is important to note that the IC_50_ required to kill the parasite is <90 nM, which is 650-fold below the concentration that would kill human cells. This confirms that Calxinin exhibits no significant toxicity in tested human cells. Additionally, no significant toxicity was detected in mice treated with high doses of Calxinin. Further testing of hematological parameters (i.e., blood homeostasis) showed that Calxinin had no effect on blood clot formation and did not alter the agonist-induced aggregation of human platelets. The PBMC proliferation induction by calxinin also needs to be explored further for the mechanism of action and implication on Malaria immune profiles and prognosis.

Although an investigation of the precise molecular mechanisms of Calxinin’s target modulation in parasites was beyond the scope of the work presented here, our results suggest that Calxinin exerts its effect through dysregulation of Ca^2+^ homeostasis in *Pf* parasites. Using complementary approaches, we show that pRBC treated with Calxinin exhibit a dramatic change in the internal Ca^2+^ concentration in parasites and, concomitantly, in the host RBCs but not in the uninfected RBCs. The intracellular Ca^2+^ concentration in pRBCs is higher than in uninfected RBCs [60,95,96]. Exposure to Calxinin caused an increase in Ca^2+^ concentrations in pRBCs. Non-infected RBCs did not show any change in Ca^2+^ concentration. We encountered two major variables for infected RBCs regarding Ca^2+^ concentrations: the developmental stage of the parasite and the age of the red blood cell, Each can cause varying basal Ca^2+^ concentrations in a given sample. When an infected red blood cell is exposed to Calxinin, it causes the rapid release of Ca^2+^ from the parasite into the host RBCs. This is only temporary as this excess Ca^2+^ is pumped out of the RBCs cytosol into the environment to reestablish the low intracellular Ca^2+^. Our photoconversion TEM data indicate that Calxinin causes a robust change in intracellular Ca^2+^ in *Pf* resulting in an irreversible change in calcium levels. This is consistent with the proposed mechanism of action of Calxinin. Interestingly, Ca^2+^ homeostasis perturbation is expected to negatively affect multiple cellular processes within parasites enhancing their vulnerability to other chemotherapeutics. Voltage-gated channel inhibitors are known to cause oxidative stress in target cells [97]. Antimalarial drugs also have oxidative stress as one of the major parasite death mediators [98]. Therefore, we hypothesize a possible synergy of Calxinin with other standard antimalarials which needs to be a prime focus of future studies.

**Figure 7:**
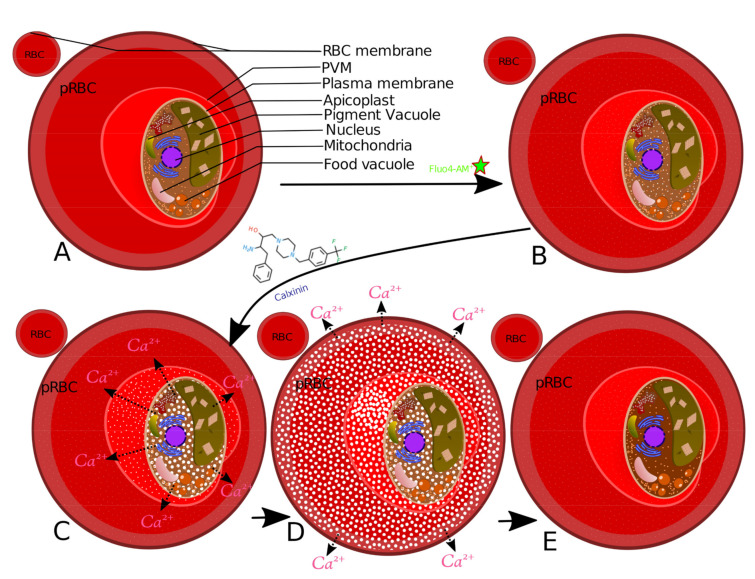
Schematic cartoon (not to scale) illustrating the observed mechanism of action of Calxinin in parasitized RBC (pRBC). Events are depicted based on both live fluorescent imaging and electron microscopy. (**A**) Two red blood cells: One is uninfected (left) and the other is a pRBC (right). The pRBC has a parasitophorous vacuolar membrane (PVM) and a trophozoite stage parasite. (**B**) An RBC is being loaded or “charged” with Fluo4-AM. The parasite, having high calcium content, fluoresces much more than the hypo-calcinated healthy and young RBCs (Figure 5A–C). Additionally, pRBCs inherently have a higher calcium level compared to RBCs. (**C**) After the addition of Calxinin, there is an initial Ca^2+^ surge within the parasite cytoplasm suggesting an organelle as the source of Ca^2+^ (Figure 5K). (**D**) The second phase of the Ca^2+^ surge causes the whole RBC to fluoresce brightly due to saturation of parasite cytoplasm and leaking of Ca^2+^ to the pRBC cytoplasm. (**E**) The surge or “spike” in pRBC Ca^2+^ rapidly comes down, while the intra-parasite Ca^2+^ level remains saturated due to continued activation by Calxinin. Total parasite Ca^2+^ normalizes over time due to the irreversible loss of Ca^2+^ (Figure 6).

Based on the live imaging and electron microscopic data the parasite, in the presence of Calxinin, exhibits an initial increase in the concentration of free Ca^2+^ in its cytoplasm. As stated above, this produces a subsequent release of Ca^2+^ from the other intracellular compartments, and this Ca^2+^ is recaptured by these compartments and/or is extruded. In the continuous presence of Calxinin, the recaptured Ca^2+^ is released again and those released cations are extruded again. This process produces the eventual emptying of the internal Ca^2+^ compartments and the translocation of the released Ca^2+^ to the external environment. This ultimately results in parasites with lower than normal Ca^2+^ concentrations near the parasite cellular peripheral zone, where acidocalcisomes are pushed by larger organelles [99]. Additionally, it should be noted that the young parasites (i.e., early ring stage) have not accumulated enough Ca^2+^ to show a robust Calxinin-induced increase in intracellular Ca^2+^ levels. The most robust Calxinin-induced increase in intracellular Ca^2+^ was observed in mid-and late-stage trophozoites infecting comparatively younger RBCs. Notably, the maximum Ca^2+^ loss was in merozoites/schizonts, which was not documented in live imaging. We interpret this finding as evidence that the Ca^2+^ surge originates from the parasite’s intracellular compartments The data reported herein, coupled with the computationally predicted target allows us to hypothesize that Calxinin directly affects a putative Ca^2+^ permeable channel, possibly the putative TRP-ML channel of *Plasmodium*. In parasitized pRBCs, Calxinin produces an increase in parasite cytoplasmic Ca^2+^ levels. This spike is masked by the high baseline level of intracellular Ca^2+^ in the parasites. In infected RBCs that are not near the maximum level of intracellular Ca^2+^, Calxinin produced a more robust transient increase in RBC intracellular Ca^2+^ which decreases gradually due to Ca^2+^ efflux into the host cell (Figure 5A–C). This could be due to a release of Ca^2+^ from the parasite’s internal compartments to the cytoplasm of the infected RBCs or by an influx of Ca^2+^ produced by Calxinin only in the infected RBCs (hypothesis depicted in Figure 7). We observed that Calxinin produced a biphasic increase in intracellular Ca^2+^ concentration. That is, Calxinin produced an increase in Ca^2+^ concentration (within parasite cytoplasm (Figure 5J and Figure 7C) followed by an increase in Ca^2+^ concentration in the infected RBC cytoplasm (Figure 5J and Figure 7D). We hypothesize that this may be due to an effect of the drug on a Ca^2+^ permeable channel, possibly the putative TRP-ML Plasmodium channel, which may reside in any of the intracellular compartments of the parasite, causing the release of Ca^2+^ into the cytoplasm of the parasite (first phase, Figure 5J and Figure 7C). This increase in the cytoplasmic Ca^2+^ concentration could, in turn, produce the release of Ca^2+^ from one or more of the other intracellular compartments (second phase, Figure 5J and Figure 7C). At the same time, the Ca^2+^extrusion mechanisms of the parasites would actively transport Ca^2+^out of the parasite causing the increase of Ca^2+^ in the cytoplasm of infected RBCs, where the action of the RBC Ca^2+^pump would transport Ca^2+^ across the RBC plasma membrane (Figure 7E). The irreversible loss of Ca^2+^ may be due to two-phase shunting acting as a one-way valve.

## 5. Conclusions

Here, we report on a novel antimalarial agent ‘Calxinin’ with a simple structure and desirable chemical properties. Calxinin was effective at nanomolar concentrations against all stages of the malaria life cycle. It was effective against common drug-sensitive and drug-resistant laboratory strains, as well as against *Pf* field isolates. Calxinin did not show adverse effects on blood coagulation dynamics or other toxicity in human cells and mice. Computational target identification and experimental validation assays revealed that Calxinin modulated Ca^2+^ levels in the plasmodium parasite. While Calxinin is structurally similar to known T-type (human) channel blockers, it is highly selective for the parasite and does not show cross-reactivity with human T-type channels. The results from live imaging support the nanomolar efficacy of the compound. We hypothesize that the RBC plasma membrane rapid Ca^2+^ efflux acts as a one-way valve, as the parasite cannot recover lost Ca^2+^ quickly due to a low Ca^2+^ concentration within the RBCs. Thus, the nanomolar concentration of Calxinin can rapidly and irreversibly deprive the parasite of critical intracellular Ca^2+^. The drug-target relationship may be further exploited to develop highly potent antimalarial agents.

Overall, Calxinin is a novel and target-based anti-malarial agent and the data presented herein suggest that it is a promising lead antimalarial compound. 

## Figures and Tables

**Table 1:** Antiparasitic IC_50_ breakpoints of Calxinin among different stages and against *Pf* and *Pb* in vitro and in vivo models. Mean IC_50_ (M ± SD) values of 3 biological replicates, and each replicate has like 10 dose concentration samples/treatments (total number of samples *n* = 30/treatment).

Strain	Stage		Host	Efficacy
*Pf*3D7	In vitro	Erythrocytic	Human RBCs	IC_50_ = 90.0 ± 1.9 nM
*Pf*DD2	In vitro	Erythrocytic	Human RBCs	IC_50_ = 88.0 ±1.1 nM
*Pf*IPC	In vitro	Erythrocytic	Human RBCs	IC_50_ = 93.0 ± 4.9 nM
*Pf*3D7	In vitro	Gametocytic	Human RBCs	50% viability loss and 100% mature gametocyte loss at 500 nM for 48 h
*Pb*ANKA	In vivo	Erythrocytic	Mice	Single 50mg/kg dose = 27.4% reduction in parasitemia
*Pb*ANKA	In vitro	Liver stages	HepG2	79.0 ± 1.6 nM
*Pb*ANKA	Ex vivo	Ookinete	Mice	Ookinete development IC_50_ = 150.0 ± 0.24 nM
*Pf* field strains	In vitro	Erythrocytic	Human RBCs	IC_50_ = 135.0 ± 6.7 nM

## Data Availability

Not applicable.

## Supplementary Materials

The following supporting information can be downloaded at: https://www.mdpi.com/article/10.3390/pharmaceutics14071371/s1, Figure S1. Multiple sequence alignment (MSA) segments of PF3D7_1313500 homologs encompassing the Calxinin binding site and pore region from different intracellular parasitic protozoa. Figure S2. Effect of Calxinin on the human T-type calcium channel. Figure S3. In vitro effects of Calxinin on human blood hemostatic parameters.

## Abbreviations

Hydroxyethylamine (HEA); dihydroartemisinin (DHA); transient receptor potential, the mucolipin (TRP-ML); *P. falciparum* (*Pf*); *P. berghei* (*Pb*); parasitized RBCs (pRBCs); calcium (Ca^2+^); Artemisinin-based combination therapies (ACTs); mechanisms of action (MOA); peripheral blood mononuclear cells (PBMCs); red blood cells (pRBCs) Chloroquine (CQ); Lumefantrine (LUM); MD (Molecular dynamic); 50% inhibitory concentration (IC_50_); Dimethyl sulphoxide (DMSO); 50% Cytotoxicity concentration (CC_50_); Structure-Activity Relationship (SAR); Optical density (OD).
