# Supplementary material for: The Multistage Antimalarial Compound Calxinin Perturbates P. falciparum Ca2+ Homeostasis by Targeting a Unique Ion Channel
Source: Pharmaceutics. 2022 Jun 28;14(7):1371. doi: 10.3390/pharmaceutics14071371 (PMC9319510; doi:10.3390/pharmaceutics14071371)
Supplement: Supplementary file 1 [file pharmaceutics-14-01371-s001.zip › pharmaceutics-1662203-supplementary.pptx]

## Slide 1
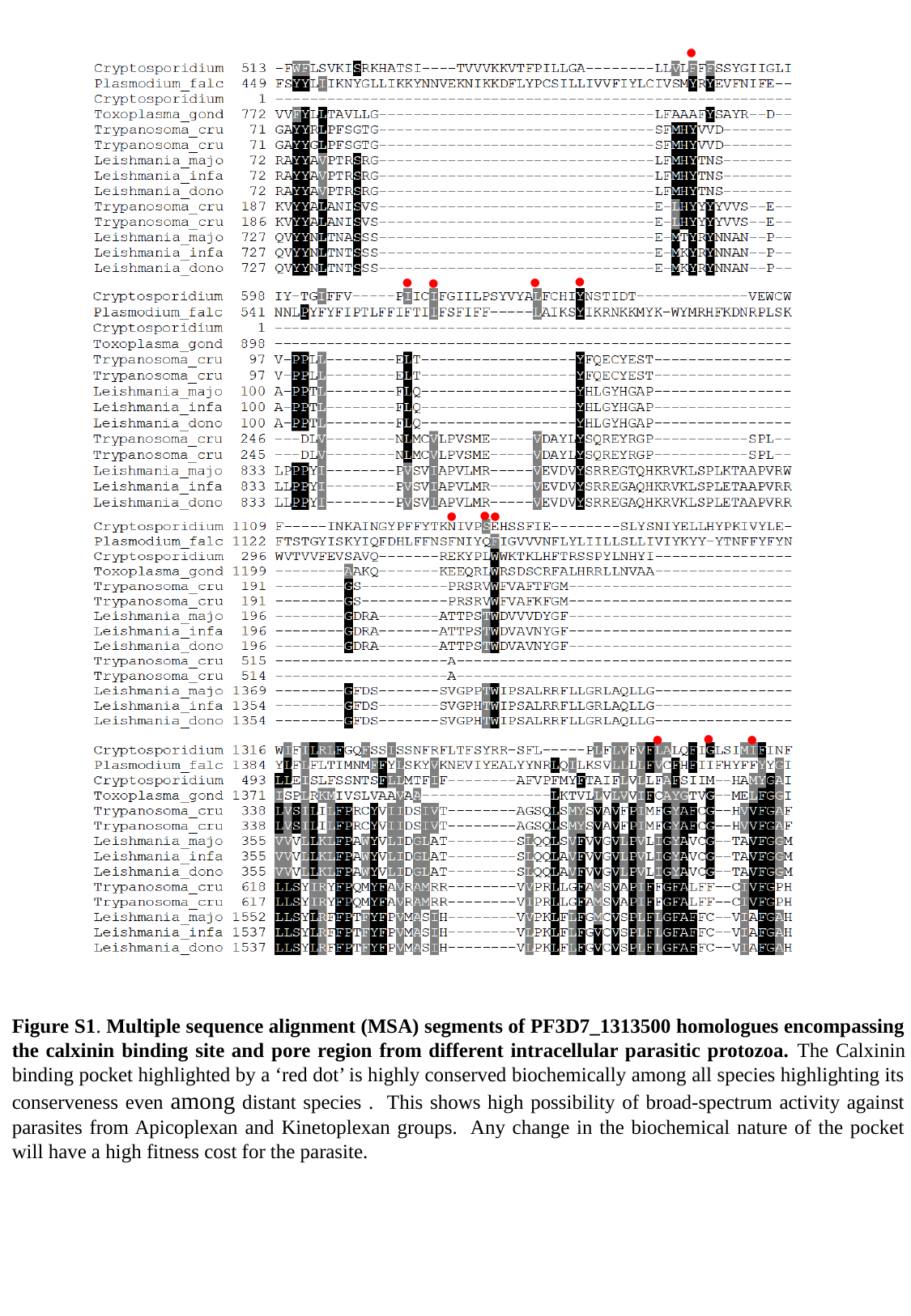

●
●
●
●
●
●
●
●
●
●
●
Figure S1. Multiple sequence alignment (MSA) segments of PF3D7_1313500 homologues encompassing the calxinin binding site and pore region from different intracellular parasitic protozoa. The Calxinin binding pocket highlighted by a ‘red dot’ is highly conserved biochemically among all species highlighting its conserveness even among distant species . This shows high possibility of broad-spectrum activity against parasites from Apicoplexan and Kinetoplexan groups. Any change in the biochemical nature of the pocket will have a high fitness cost for the parasite.

## Slide 2
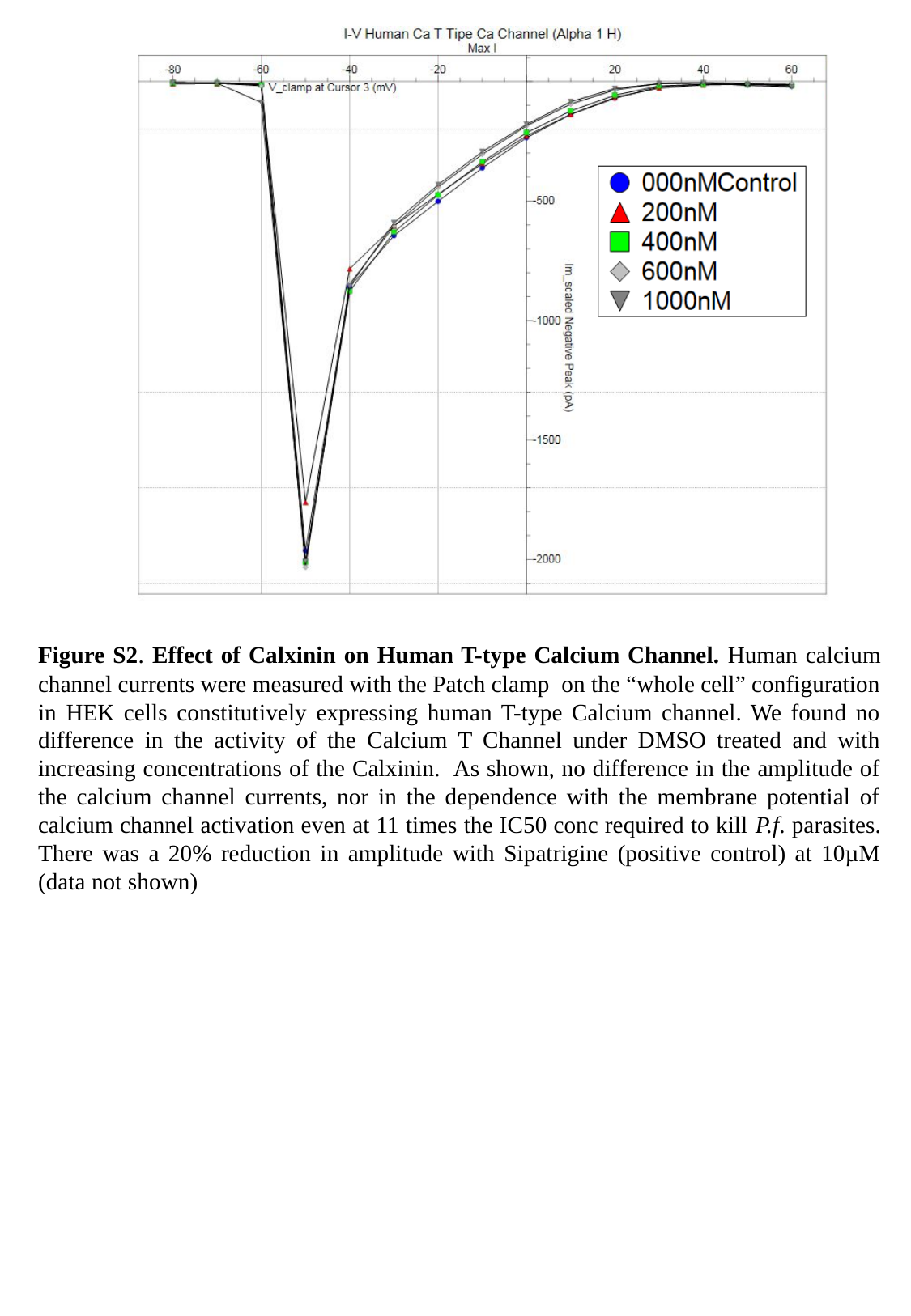

Figure S2. Effect of Calxinin on Human T-type Calcium Channel. Human calcium channel currents were measured with the Patch clamp on the “whole cell” configuration in HEK cells constitutively expressing human T-type Calcium channel. We found no difference in the activity of the Calcium T Channel under DMSO treated and with increasing concentrations of the Calxinin. As shown, no difference in the amplitude of the calcium channel currents, nor in the dependence with the membrane potential of calcium channel activation even at 11 times the IC50 conc required to kill P.f. parasites. There was a 20% reduction in amplitude with Sipatrigine (positive control) at 10µM (data not shown)

## Slide 3
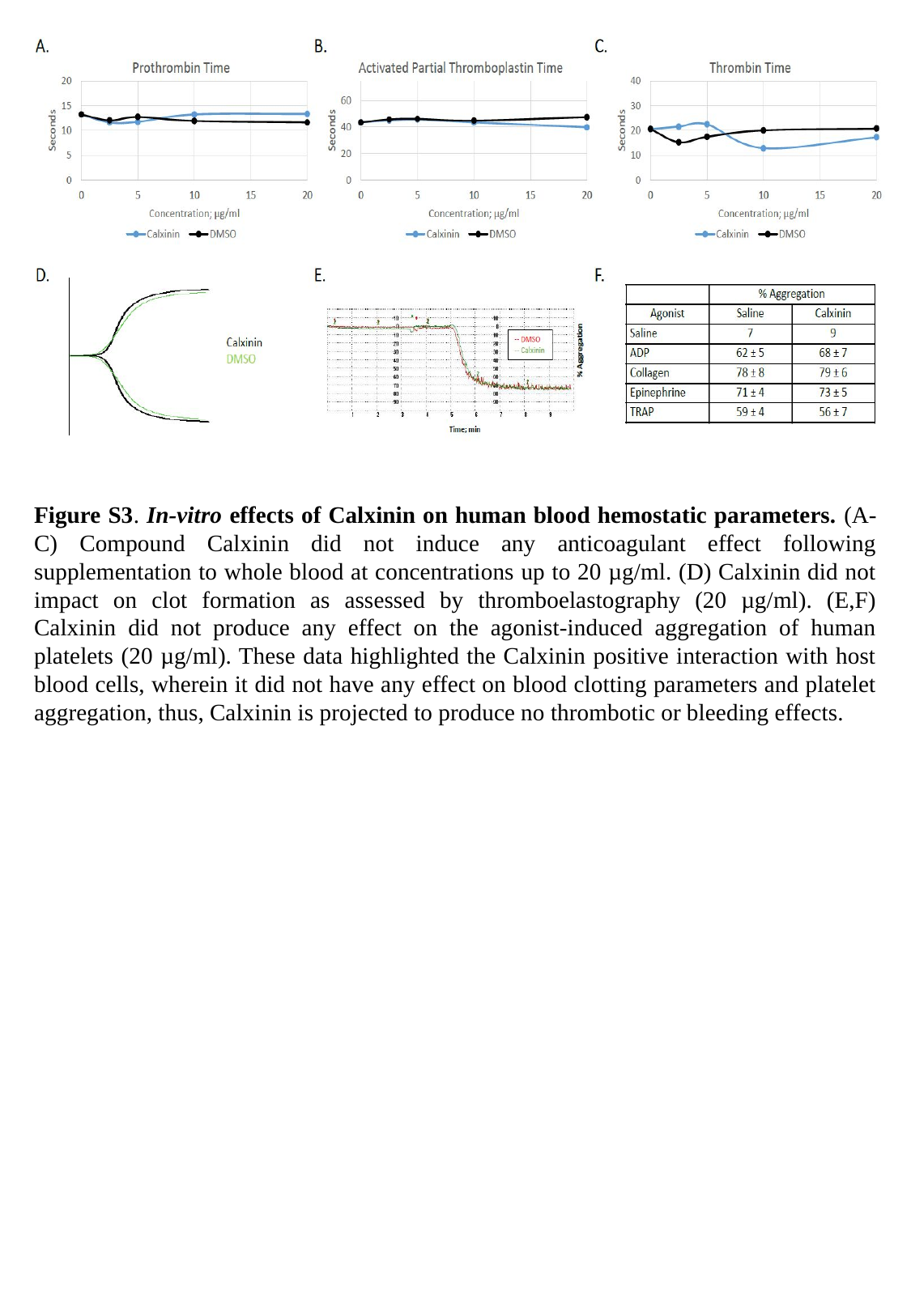

#
Figure S3. In-vitro effects of Calxinin on human blood hemostatic parameters. (A-C) Compound Calxinin did not induce any anticoagulant effect following supplementation to whole blood at concentrations up to 20 µg/ml. (D) Calxinin did not impact on clot formation as assessed by thromboelastography (20 µg/ml). (E,F) Calxinin did not produce any effect on the agonist-induced aggregation of human platelets (20 µg/ml). These data highlighted the Calxinin positive interaction with host blood cells, wherein it did not have any effect on blood clotting parameters and platelet aggregation, thus, Calxinin is projected to produce no thrombotic or bleeding effects.
